# Spatio-temporal landscape of mouse epididymal cells and specific mitochondria-rich segments defined by large-scale single-cell RNA-seq

**DOI:** 10.1038/s41421-021-00260-7

**Published:** 2021-05-18

**Authors:** Jianwu Shi, Kin Lam Fok, Pengyuan Dai, Feng Qiao, Mengya Zhang, Huage Liu, Mengmeng Sang, Mei Ye, Yang Liu, Yiwen Zhou, Chengniu Wang, Fei Sun, Gangcai Xie, Hao Chen

**Affiliations:** 1 Institute of Reproductive Medicine, Medical School of Nantong University, Nantong, Jiangsu 226019 China; 2 School of Biomedical Sciences, Faculty of Medicine, The Chinese University of Hong Kong, Hong Kong, SAR China; 3 Department of Plastic and Reconstructive Surgery, Shanghai Ninth People’s Hospital, Shanghai Jiao Tong University School of Medicine, Shanghai 200011, China

**Keywords:** Bioinformatics, Gene expression profiling, Transcriptomics

## Abstract

Spermatozoa acquire their fertilizing ability and forward motility during epididymal transit, suggesting the importance of the epididymis. Although the cell atlas of the epididymis was reported recently, the heterogeneity of the cells and the gene expression profile in the epididymal tube are still largely unknown. Considering single-cell RNA sequencing results, we thoroughly studied the cell composition, spatio-temporal differences in differentially expressed genes (DEGs) in epididymal segments and mitochondria throughout the epididymis with sufficient cell numbers. In total, 40,623 cells were detected and further clustered into 8 identified cell populations. Focused analyses revealed the subpopulations of principal cells, basal cells, clear/narrow cells, and halo/T cells. Notably, two subtypes of principal cells, the Prc7 and Prc8 subpopulations were enriched as stereocilia-like cells according to GO analysis. Further analysis demonstrated the spatially specific pattern of the DEGs in each cell cluster. Unexpectedly, the abundance of mitochondria and mitochondrial transcription (MT) was found to be higher in the corpus and cauda epididymis than in the caput epididymis by scRNA-seq, immunostaining, and qPCR validation. In addition, the spatio-temporal profile of the DEGs from the P42 and P56 epididymis, including transiting spermatozoa, was depicted. Overall, our study presented the single-cell transcriptome atlas of the mouse epididymis and revealed the novel distribution pattern of mitochondria and key genes that may be linked to sperm functionalities in the first wave and subsequent wave of sperm, providing a roadmap to be emulated in efforts to achieve sperm maturation regulation in the epididymis.

## Introduction

The epididymis is a critical male sex organ that plays key roles in sperm transport, maturation, and storage^1,2^. Spermatozoa from the testes acquire their motility and fertilization ability when they transit through the epididymis. In the epididymis, the sperm plasma membrane is subjected to sequential biochemical and proteomic modifications via interactions with components of the extracellular environment in the epididymal lumen.

The epididymis epithelium supports a luminal environment that promotes sperm maturation, and each of the three regions—caput, corpus, and cauda—is believed to play a distinctive role during sperm transition^3,4^. Luminal secretions from the caput and corpus are beneficial for the acquisition of sperm motility and fertilizing ability^5^. On the other hand, caudal epididymal secretions maintain the condition for the storage of spermatozoa while preserving their fertility^6^. Each of these regions has been demonstrated to possess a distinct pattern of gene expression related to specific physiological functions that are important in the different steps of sperm maturation^7–10^.

The composition of the intraluminal milieu is controlled by the surrounding pseudostratified epithelium, which is composed of multiple cell types possessing distinct physiological functions, including principal cells, basal cells, and clear/narrow cells^2,11,12^. In addition, a few studies have reported apical cells and halo cells in the epididymis^13^, despite the lack of specific markers to identify them. Studies of region-specific epididymal proteins showed that certain cell types were able to express different classifications of genes, which contribute to the different physiologic functions of the segments^2,14^. With the development of single-cell RNA sequencing (scRNA-seq), a number of organs have been analyzed in mammals^15–17^, including male and female reproductive organs such as the testis^18–20^ and ovary^21,22^. The spatio-temporal repertoire of epididymal cells and their gene expression in the epididymis are still less characterized.

In this study, we applied microfluidic-based scRNA-seq to analyze the cells derived from the caput, corpus, and cauda of the mouse epididymis aged 42 days (P42) and 56 days (P56), representing the first wave and subsequent wave of sperm maturation, respectively^23^. In total, 40,623 cells were analyzed at the large-scale number level, and further clustering analysis revealed eight cell types. Furthermore, the subpopulations of epididymal epithelial cells were described, and two subtypes of principal cells with cilia were identified. We further demonstrated the segment-specific and cell-type-specific gene expression patterns of epididymal cells. Remarkably, the number of mitochondria and their corresponding mitochondrial transcripts (MTs) were found to be higher in the corpus and cauda than in the caput of the epididymis in both the P42 and P56 epididymis. In addition, the expressed gene comparison of the first-wave sperm P42 and adult sperm P56 was undertaken at the single-cell level. Overall, our study represents the regional transcriptome profiling of the mouse epididymis at a single-cell resolution, which is important for future studies on the spatial microenvironments of the epididymis and the understanding of sperm maturation and epididymal diseases.

## Results

### ScRNA-seq analyses revealed comprehensive epididymal cell types

**Fig. 1:**
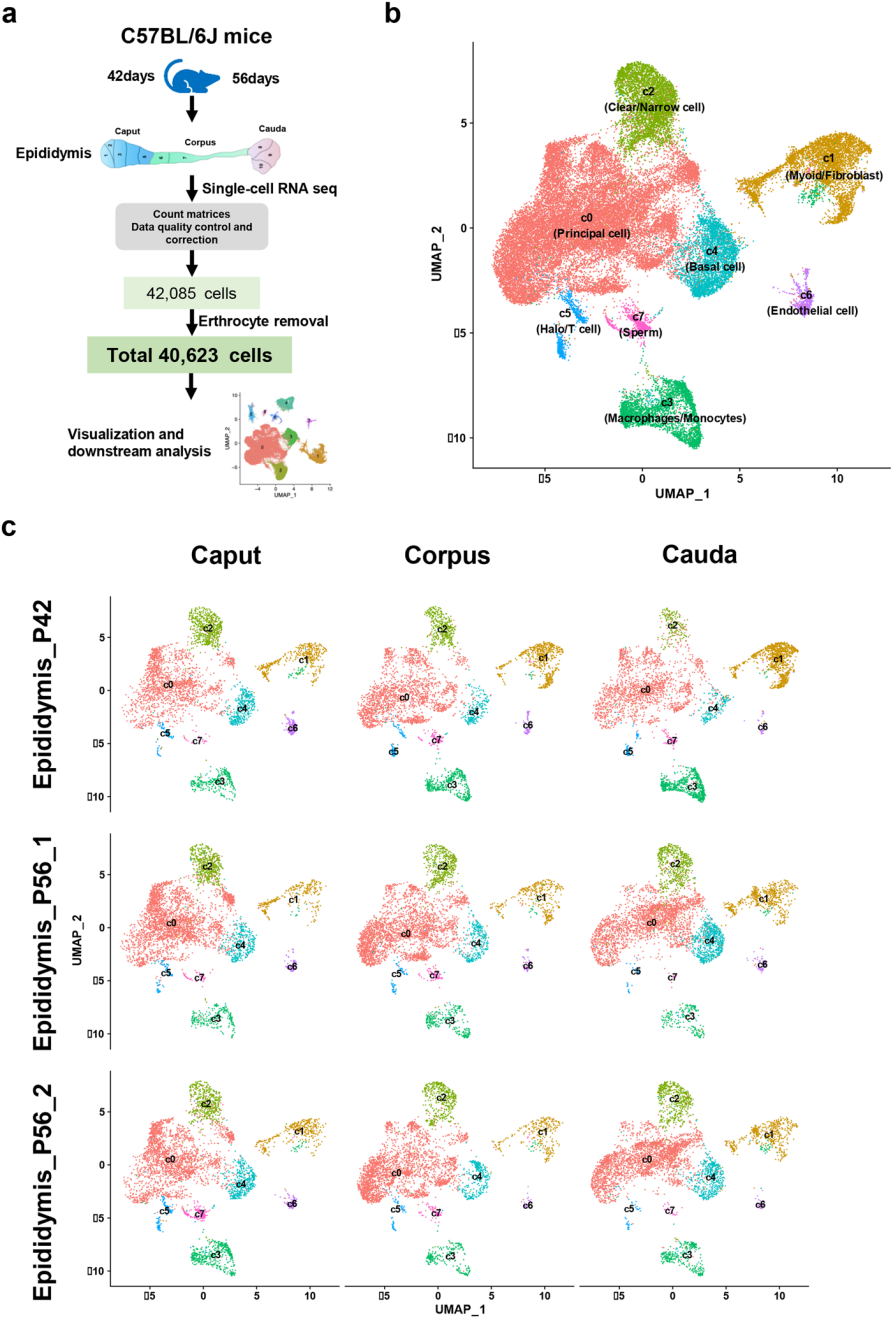
Overview of scRNA sequencing of mouse epididymis regions. **a** Schematic of mouse epididymis collection and analysis. **b** UMAP visualization of the eightcell clusters identified in the epididymis after erythrocyte filtration. **c** UMAP visualization of the three regions of the mouse epididymis in P42 and P56 mice. Each epididymal region of the P56 epididymis has two biological replicates.

To define the cell types in the mouse epididymis, we conducted scRNA-seq on the cells isolated from fresh caput, corpus, and cauda regions of the epididymides at two key developmental times: 13,360 cells from the 42-day-old epididymis that contained the first wave spermatozoa and 28,725 cells from the 56-day-old epididymis in two biological replicates (Fig. 1a and Supplementary Fig. S1a). Following cell quality control (“Materials and Methods” section and Supplementary Table S3), a total of 42,085 cells from the three regions of the epididymis were visualized and identified into nine cell clusters (Supplementary Fig. S1b, c). We first annotated the cell types using the reported cell markers according to the literature (Supplementary Fig. S1d, e and Table S1). Cells in cluster 5 highly expressed Hba and Hbb and were identified as erythrocytes, which were generally excluded from previous studies^24–26^. Therefore, cell cluster 5 was excluded from all subsequent analyses, and the number of remaining cells was 40,623. We assigned eight cell populations in all segments of the epididymis (Fig. 1b, c) in the P42 and P56 epididymis through Seurat v3. Cell clusters were annotated as principal cells (C0), myoid cells/fibroblasts (C1), clear/narrow cells (C2), macrophages/monocytes (C3), basal cells (C4), halo/T cells (C5), endothelial cells (C6) and sperm (C7) (Fig. 1b and Supplementary Fig. S2).

### scRNA-Seq analyses recapitulate subpopulations of principal cells, basal cells, and clear cells

**Fig. 2:**
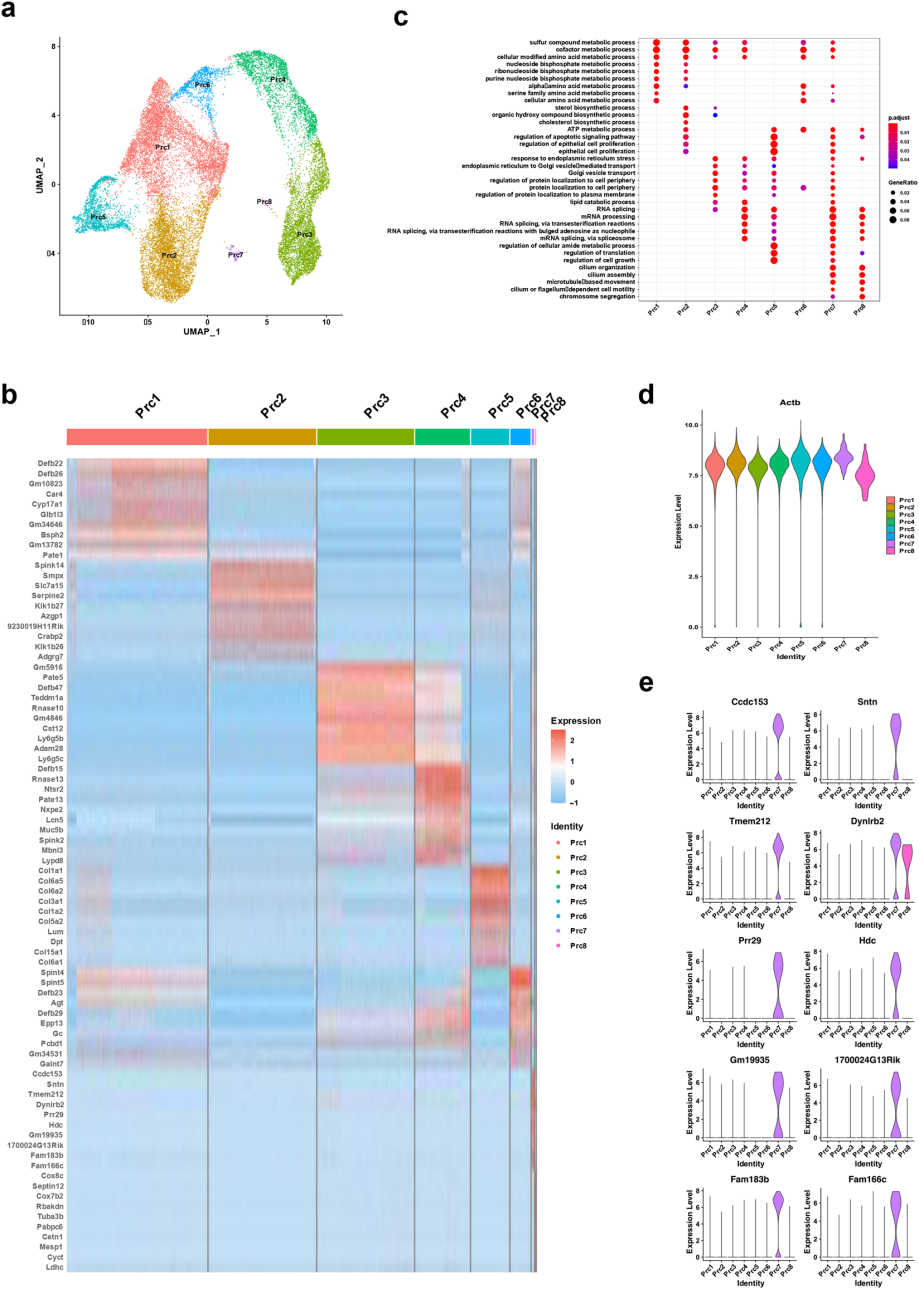
Features of principal cell subpopulations. **a** UMAP representation of the subpopulations of principal cells aligned in the caput, corpus, and cauda. Eight subclusters of principal cells were identified. **b** Heatmap showing the top 10 marker genes for each subpopulation (log1pRPM scaled by row). **c** GO enrichment analysis for the principal subpopulation. **d** Violin plot of actin (Actb) in the principal subpopulation (*y*-axis log1pRPM). The expression level of Actb was the highest in the Prc7 subcluster. **e** Violin plot showing representative marker genes for the principal subpopulation Prc7 (*y*-axis log1pRPM).

To clarify the subpopulations in principal cells, we employed a higher SNN clustering resolution. Eight different subpopulations of principal cells were revealed from the caput to cauda, which were defined as Prc1-Prc8 (Fig. 2a). The top 10 differentially expressed genes (DEGs) were illustrated in each of the eight subpopulations (Fig. 2b). Furthermore, the GO terms associated with the DEGs in each cell cluster (Fig. 2c) manifested diverse roles of the principal cells in regulating the epididymal microenvironments. Intriguingly, genes enriched in Prc7 and Prc8 were related to cilium organization and assembly, microtube-based movement and cilium-dependent cell motility according to GO analysis (Fig. 2c). These two clusters of principal cells were consistent with histologically described epididymal cells with stereocilia accompanying the expression of actin (Actb)^27,28^. Although the expression of Actb was highest in the Prc7 subpopulation, it was also expressed in other subpopulations of principal cells (Fig. 2d). Therefore, we plotted the potential marker genes of Prc7 principal cell subsets and visualized them in violin plots (Fig. 2e).

In addition to principal cells, the subpopulations of other major epididymal epithelial cells were also examined. In detail, four subtypes of cells were identified in each cluster of basal cells, clear cells, and halo cells (Supplementary Figs. S3–S5). The DEGs and GO term enrichments for each subpopulation of epididymal cell clusters are listed in Supplementary Tables S6 and S7. These results highlight the complex transcriptional regulation patterns in epididymal subpopulations.

To further examine the interactions among the different cell clusters, cell–cell communications were studied using CellTalkDB and SingleCellSignalR. As shown in Supplementary Fig. S6, wide paracrine communications between each pair of cell types were discovered (Supplementary Fig. S6a). Interestingly, nonepithelial cells such as endothelial cells (C6) and macrophages/monocytes (C3) were able to affect epididymal epithelial cells, including principal cells, clear/narrow cells, and basal cells, through several specific genes (Supplementary Fig. S6b–h). In particular, the potential pairs of cells in communication via ligand receptors were deciphered—for example, communication from endothelial cells to clear/narrow cells (Supplementary Fig. S6b), principal cells (Supplementary Fig. S6c), and basal cells (Supplementary Fig. S6d). In addition, macrophages/monocytes were found to influence other cell clusters, such as principal cells (Supplementary Fig. S6e), clear/narrow cells (Supplementary Fig. S6f), and basal cells (Supplementary Fig. S6g). Of note, ligand-receptor pairs, including Ccl5-Ackr1 and Gnai2-Cnr1, were observed in all cell-cell communications between macrophages/monocytes and other cell types. In addition to intercellular communication between nonepithelial cells and epithelial cells, the interplay between basal cells and principal cells via Hsp90b1-Tlr9 and App-Drd3/scrcs1 was also observed (Supplementary Fig. S6h). Detailed information on the ligand-receptor interactions for each cell pair is listed in Supplementary Table S8. These results indicate the complex cell-cell interaction among the epididymal epididymis.

### Segment characterization of gene expression and mitochondrial signatures in the epididymis

**Fig. 3:**
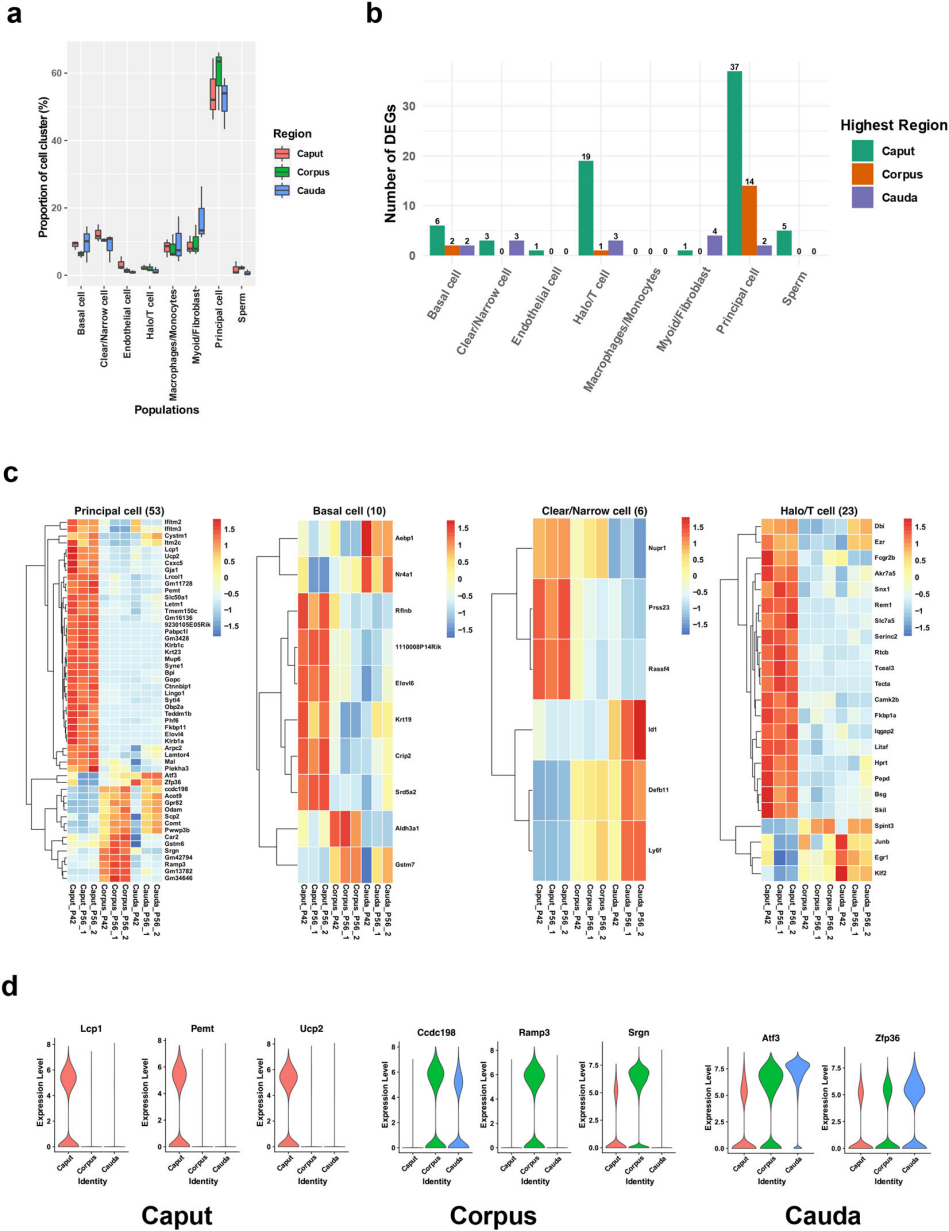
Segmental DEGs of the mouse epididymis. **a** The spatial proportion of each cell cluster is illustrated. **b** The distribution of the number of segmental DEGs in each cell cluster. **c** Heatmaps of DEG comparisons in three epididymal regions from the epididymal epithelial cell clusters (log1pRPM scaled by row). **d** Representative spatially specific DEGs of principal cells in three epididymal segments (*y*-axis log1pRPM).

In our analysis, the proportions of cell clusters were similar in the caput, corpus, and cauda epididymis. Consistent with a previous study^2^, the percentage of principal cells was ~60% in the three epididymal regions (Fig. 3a). Intriguingly, the number of segmental differentially expressed genes (DEGs) was consistently higher in the caput epididymal cells (Fig. 3b). It is well known that the expression of epididymal genes shows segment-specific or distally changed patterns in the epididymis^29,30^. To further elucidate the segment specificity in the context of gene expression profiles, we compared the DEGs in all cell types along with the three segments, and representative heatmaps are shown in Fig. 3c and Supplementary Fig. S7a. GO enrichment revealed the divergent roles of each cell type (Supplementary Fig. S7b). The representative segmental-specific genes in principal cells are illustrated in Fig. 3d.

**Fig. 4:**
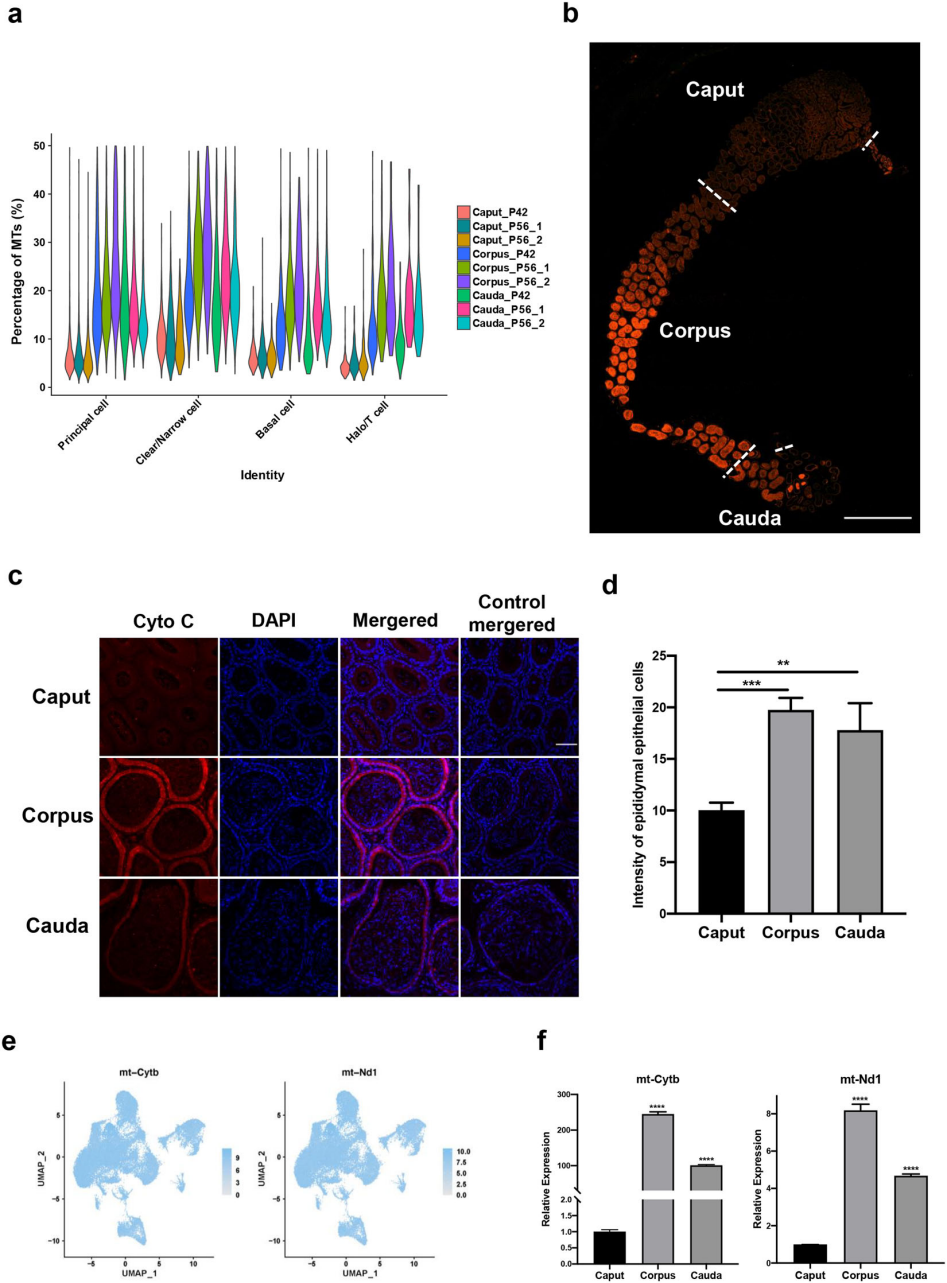
Characterization of the mitochondrial distribution in epididymal regions. **a** The segmental percentage of mitochondrial transcripts (MTs) in epididymal epithelial cell clusters. **b** Stitched image of immunostaining of the mitochondrial marker cytochrome c in the mouse epididymis. Scale bar = 1 mm. **c** Representative images of cytochrome c staining in the caput, corpus and cauda epididymis. Scale bar = 50 µm. Red: cytochrome c (cyto C), Blue: DAPI for nuclear count staining. **d** Corresponding statistics of cytochrome c staining in the segmental epididymis. **P < 0.01; ***P < 0.001. **e** UMAP plots of mitochondrial transcript genes (mt-cytb and mt-Nd1) in the indicated cell clusters (color values were log1pRPM). **f** Representative mitochondrial transcript genes (mt-cytb and mt-Nd1) in sperm-depleted epididymal segments by qPCR analysis. The statistical significance was corpus or cauda *vs.* caput. ****P < 0.00001.

Unexpectedly, a consistently higher percentage of mitochondrial transcripts (MTs) was observed in the corpus and cauda epididymis than in the caput (Supplementary Fig. S1b, f). Based on the P42 epididymis results, an average of 7.7% of UMIs was MTs in the caput segment, while those in the corpus and cauda segments were 17.0% and 13.7%, respectively (Supplementary Table S9). Such higher MT percentages in corpus and cauda segments were reproducibly observed in two replicates of the P56 epididymis, which showed 20.4–25.6% and 16.5–18.8% MTs in the corpus and cauda segments, respectively (Supplementary Table S9). Consistent with observations in the P42 caput epididymis, 7.1–7.5% MTs were found in the caput segment of the P56 epididymis (Supplementary Table S9). Furthermore, MT transcript percentages in four epididymal cell populations (principal cells, basal cells, clear/narrow cells, and halo/T cells) were analyzed, and high MT percentages were observed in the corpus and cauda segments, as well as in the global analysis (Fig. 4a).

To confirm our scRNA-seq observation, we used mitochondrial tracker staining and immunofluorescence staining to examine the distribution of mitochondria in epididymal epithelial cells. Indeed, either the mitochondrial-specific marker cytochrome c (Figs. 4b–d, and Supplementary Fig. S8b) or mitochondrial tracker staining of CMXRos (Supplementary Fig. S8a and S9) showed a significantly higher mitochondrial number in the corpus and cauda than in the caput epididymis in both P42 and P56 mice. In addition, we validated the mRNA expression levels of selected mitochondrial genes by qPCR. In line with the scRNA-seq results, the mitochondrial genes mt-cytb, mt-ND1-6, mt-Co1, and mt-Rnr1-2 were highly expressed in the corpus and cauda epididymis (Fig. 4e, f, and Supplementary Fig. S10). A further result of KEGG pathways analysis was enriched in Supplementary Fig. S11.

### Transcriptomic variation in the first wave and adult epididymal sperm

**Fig. 5:**
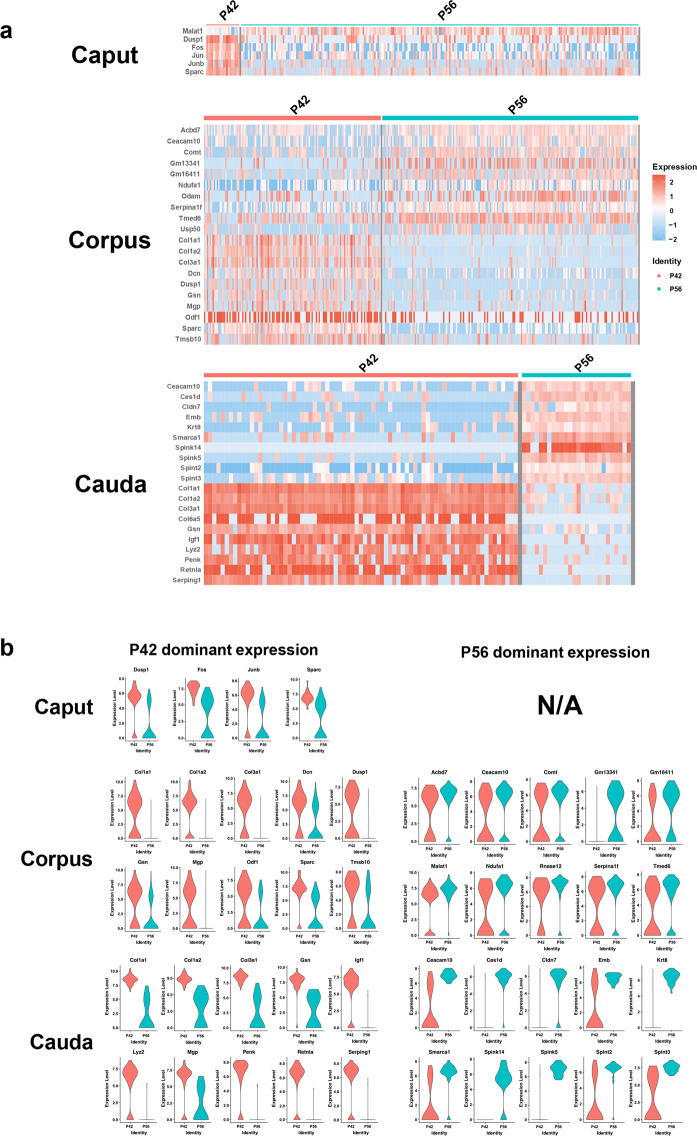
Segmental DEG comparison of first-wave sperm (P42) production and adult sperm (P56). **a** Heatmaps of DEGs for P42 and P56 spermatozoa in the caput, corpus, and cauda epididymis (log1pRPM scaled by row). **b** Violin plots showing segmental expression of spermatozoa DEG genes at different stages. NA: There were no representative DEGs that were highly expressed in P56 sperm compared to P42 sperm in the caput epididymis.

Although sperm are transcriptionally and translationally quiescent in the traditional concept, emerging evidence has shown that sperm have transcriptional potential because of the presence of histone proteins and reverse transcriptase activity in mature spermatozoa^31–33^. By using robust scRNA-seq technology, we compared the transcriptomes of the first-wave sperm (P42) and adult sperm (P56) in the mouse epididymis. We first examined the predominant expression of DEGs in two developmental stages (P42 vs. P56) in the caput, corpus, and cauda segments (Fig. 5a and Supplementary Fig. S12), illustrating the temporal divergence of the epididymis. In particular, the gene expression levels in P42 spermatozoa were higher than those in P56 spermatozoa in the caput epididymis. The top 10 genes predominantly expressed in P42 or P56 epididymal spermatozoa are plotted in Fig. 5b to illustrate the differences in sperm between the two developmental stages. These differences suggest a differential regulatory mechanism underlying epididymal sperm maturation in the first wave *vs.* the subsequent wave of sperm.

## Discussion

The mammalian epididymis is composed of convoluted and interconnected regions, each of which contains a complex mosaic of spatially intermixed cells. Accumulating studies have attempted to characterize the cell types in the epididymis based on cell location, morphology, connection, function, and marker gene expression^34–36^. A scRNA-seq dataset from the mouse genital duct was recently reported^37^. While our results are consistent with a previous report on the clustering of general cell types, our study provided an in-depth analysis of biological replicates. Notably, our study also uncovers the segmental distribution of mitochondria and mitochondrial transcripts using a low threshold of mitochondrial reads. Furthermore, we revealed the clustering of epididymal cells with stereocilia and compared the epididymis at different developmental stages. It should be noted that sperm were not depleted from the epididymis in our study. Thus, the scRNA-seq dataset may represent the physiological microenvironments in which sperm continuously transit in the epididymis. Therefore, caution should be taken when comparing the different datasets on the caput and corpus epididymis. Unfortunately, a direct comparison between our data and an available scRNA-seq dataset of human epididymal caput was not feasible since only the proximal epididymis was reported^38^.

Over the past years, we have elucidated the functions of a number of epididymal proteins in rodents to advance the understanding of sperm maturation^39–45^. In this study, we constructed the cell landscape of the mouse epididymis with recognized segment separation^7,46,47^ (Fig. 1a and Supplementary Fig. S1a). In total, we identified eight epididymal cell populations from 40,623 cells. In corroboration with previous studies, we found four known cell types: principal cells (C0), clear/narrow cells (C2), basal cells (C4), and halo cells (T cells) (Fig. 1). These cell types are the main components of the epididymal epithelium. Principal cells are the most abundant cell type in the epididymal epithelium and have multiple functions, including the modulation of proton secretion and thus the luminal pH and the secretion of a plethora of proteins, small RNA, and extracellular vesicles into the epididymal lumen for sperm maturation^2,30^. Compared to the principal cells, the functions of other cell types are less known. Clear/narrow cells were reported to be involved in proton secretion with the expression of H^+^-ATPase^2,48^. Basal cells were proposed to participate in the endocrine or paracrine regulation of principal cells^49^. In addition, recent studies postulate the potential role of basal cells as a stem cell compartment in the epididymis^38,50^. Halo cells (T cells) appear to be one kind of immune cell in the epididymis. Consistent with a recent study^37^, epididymal supporting cells were also identified. These include myoid cells/fibroblasts (C1) for contraction of the myoid to promote sperm movement and form an extracellular environment^2,37,51^, macrophages/monocytes (C3) for immune equilibrium^52^, and endothelial cells (C6) for composing blood and lymph vessels^37^. In addition to epididymal cells, sperm (C7) were also identified because of the whole epididymis dissociation applied in our study.

Similar to other single-cell RNA-seq studies, novel subclusters of cells were also found in principal cells, basal cells, clear/narrow cells, and halo/T cells. Notably, benefitting from the larger number of epididymis single cells profiled, the subpopulation of principal cells with stereocilia was clustered at the gene expression level, which was previously described in histological sections of the epididymis^28,53,54^. The detailed characteristics and roles of Prc7 principal cells warrant further investigation.

Emerging studies have endeavored to elucidate the regulation of region-specific gene expression in the epididymis using various strategies^7,29,55–57^. Region-specific gene expression in the epididymis in mice and humans was also studied by microarray analysis^7,56^. However, these microarray studies were not compared to our dataset, as the original data were not readily available. Nonetheless, our results depicted the cell landscape of each region of the mouse epididymis and provided an in-depth analysis of the gene expression profile of each cell population. Predominantly expressed genes with segment-specific patterns in principal cells were depicted, and some of the genes were consistent with previous studies. For example, Defb41, which was enriched in caput principal cells, was found to affect sperm motility and zona pellucida binding^58^. Our results, together with previous findings, demonstrated not only the potential involvement of segment-specific genes in sperm functions but also critical cell-specific regulation primarily by principal cells during sperm maturation. In addition, the involvement of segment-distributed genes in different cell clusters, such as Spint4 and Lcns, in sperm maturation warrants further investigation.

Another important finding of this study is the high mitochondrial content in the epididymal epithelial cells of the corpus and cauda compared to the caput epididymis (Fig. 4). Mitochondria, which are a very important and abundant organelle type in the cytoplasm, are involved in various processes, such as generating adenosine triphosphate (ATP), establishing developmental competence, maintaining calcium homeostasis, and regulating apoptosis^59^. It is well known that mitochondria are critical for oocyte maturation, fertilization, and embryonic development^60,61^. In the male reproductive tract, mitochondria are involved in the regulation of spermatogenesis, sperm production, and multiple sperm functions^62,63^. Although mitochondria-rich apical cells are found in the caput epididymis^13^, few studies on mitochondrial distributions and functions of epididymal epithelial cells have been reported. In contrast with previous studies, we for the first time found that both the abundance of mitochondria and the percentage of MTs in epididymal epithelial cells were significantly increased in the corpus and cauda regions compared to the caput region of the epididymis. Of note, the percentage of MTs in the corpus epididymis reached 21%. Although a high percentage of MTs is considered an indicator of cell stress or cell death^64^, the amount of MTs varies in different tissue types, including the heart, kidney, and brain^65–67^. A recent study demonstrated that tissues including the heart, adrenal gland, and liver have higher percentages of mitochondrial RNA levels (>20%) than those of other tissues (<5%, the epididymis was not examined)^65^. In the mouse testis, the percentage of mitochondria-encoded RNAs was found to be approximately 10% in Sertoli cells^18^. Similarly, the high mitochondrial number and MT% in the epididymis may be required to provide sufficient energy for the synthesis and secretion of thousands of molecules that are required for various sperm functions.

Another aspect of our scRNA-seq analyses may illustrate the differences in the developmental time points of the epididymis and its spermatozoa. In this study, we compared the DEGs between P42 and P56 epididymal cells that cover the first-wave and adult sperm in the epididymis. We found the predominant time expression patterns in the epididymal cells along the three regions of the epididymis. Although the study demonstrated a normal fertilization capacity of sperm derived from the P42 epididymis, the analysis of our dataset suggests divergent expression profiles in both epididymal cells and sperm between the two different developmental time points.

Finally, emerging investigations have reported the possible mechanisms between the epididymal environment and sperm maturation by comparing the sperm proteome^68–70^ and sperm small RNAs^71^ from the caput to cauda. However, few studies have focused on the mRNA of spermatozoa. Xiaoxia et al. summarized recent studies on the transcripts of sperm during post-meiotic spermatids, sperm motility, sperm capacitation, and cryopreservation^72^. In line with the time-specific expression of genes in epididymal epithelial cells, we uncovered the transcript differences of sperm in P42 and P56 spermatozoa in the epididymis at the single-cell level. As expected, we also found a time-predominant expression profile when comparing P42 spermatozoa with P56 spermatozoa. Interestingly, the highly expressed genes of P56 spermatozoa mainly existed in the corpus and cauda segments, suggesting that the regulation of transcripts in caput spermatozoa was completed at the P42 epididymis. Notably, the expression of Col3a1 was higher in P42 spermatozoa than in P56 spermatozoa through the three regions of the epididymis. James et al. demonstrated that Col3a1 was differentially distributed from caput to cauda epididymal epithelial cells^73^. The role of high Col3a1 transcripts in first-wave (P42) spermatozoa needs to be further elucidated. Collectively, our single-cell dataset deciphers the discrepancies between the transcripts of first-wave and adult spermatozoa in the epididymis, although testicular factors cannot be excluded at the current stage.

Taken together, our data provide an indispensable resource at a single-cell resolution for a comprehensive cell atlas and spatio-temporal profile of the epididymis. The present results also demonstrated the elevated mitochondrial level of epididymal epithelial cells in the corpus and cauda epididymis and its potential involvement during sperm maturation. Our study has shed light on the understanding of normal epididymis functionalities and the cellular mechanisms of epididymis diseases that cause male infertility.

## Materials and Methods

### Animals and epididymis sample collection

Sample collection was carried out under license in accordance with the Guidelines for Care and Use of Laboratory Animals of China, and all protocols were approved by the Institutional Review Board of Nantong University. Five 42-day-old (P42) and ten 56-day-old (P56) C57BL/6J mice were used in this study. After sacrificing the mice, the epididymis was dissected and divided into three regions (caput, corpus, and cauda) as previously described^7,74^. Briefly, the mice were sacrificed by anesthetized cervical dislocation, and the epididymis was quickly isolated and placed in a petri dish with ice-cold saline. Epididymal fat was carefully removed, and the sample was separated into caput, corpus, and cauda regions under a dissecting microscope. The different segments of each epididymis were immediately transferred into GEXSCOPE^TM^ Tissue Preservation Solution (Singleron Biotechnologies) in Eppendorf tubes labeled EP (caput), EO (Corpus), and ED (cauda) on ice. The procedure was repeated in five mice until all segments were collected in each tube. Two replicates for each pooled segment of the P56 epididymis were prepared. The pooled segments were further processed following the procedure of tissue dissociation and single-cell preparation.

### Tissue dissociation and single-cell suspension preparation

Prior to tissue dissociation, the specimens were washed with Hanks balanced salt solution (HBSS) three times and minced into 1–2 mm pieces. The tissue pieces were digested in 2 mL GEXSCOPE^TM^ Tissue Dissociation Solution (Singleron Biotechnologies) at 37 °C for 15 min in a 15 mL centrifuge tube with continuous agitation. Following digestion, a 40-micron sterile strainer (Corning) was used to separate cells from cell debris and other impurities. The cells were centrifuged at 1000 rpm and 4 °C for 5 min, and the cell pellet was resuspended in 1 mL PBS (HyClone). To remove red blood cells, 2 mL GEXSCOPE^TM^ Red Blood Cell Lysis Buffer (Singleron Biotechnologies) was added to the cell suspension and incubated at 25 °C for 10 min. The mixture was then centrifuged at 1000 rpm for 5 min, and the cell pellet was resuspended in PBS. Cells were counted with a TC20 automated cell counter (Bio-Rad).

### scRNA sequencing library preparation

The concentration of the single-cell suspension was adjusted to 1 × 10^5^ cells/mL in PBS. A single-cell suspension was then loaded onto a microfluidic chip^75^ (part of the Singleron GEXSCOPE^TM^ Single Cell RNA-seq Kit, Singleron Biotechnologies), and single-cell RNA-seq libraries were constructed according to the manufacturer’s instructions (Singleron Biotechnologies). Briefly, a single cell suspension was loaded onto the microchip to partition single cells into individual wells on the chip. Cell barcoding beads were then loaded into the microchip and washed. Afterward, 100 μL of single-cell lysis buffer was added to the chip to lyse cells and capture mRNAs at room temperature for 20 min. The beads, together with the captured RNAs, were flushed out of the microchip and used for subsequent reverse transcription, cDNA amplification, and library construction. After size selection and purification, the scRNA-seq libraries were sequenced on an Illumina HiSeq ×10 instrument with 150-bp paired-end reads.

### scRNA-seq alignment and UMI calculation

The raw sequencing data were preprocessed before analysis via the following procedures: data preprocessing, cell barcode extraction, genomic read alignment, and unique molecular identifier (UMI) counting. In detail, first, reads with low sequencing qualities were filtered, and the sequencing adapters were trimmed by Fastp^76^ software (fastp 0.19.5) using the default settings. Then, umi_tools^77^ was used to identify and extract cell barcodes with the settings of a cell number of 5000 and an error correction threshold of 2. Next, STAR genomic mapper was applied to map the extracted reads to the mouse Gencode genome (GRCm38.primary_assembly.genome.fa, version M18). FeatureCounts was used to assign exon-level reads based on Gencode gene annotation (gencode.vM25.primary_assembly.annotation.gtf, version M25). Furthermore, the UMIs for each gene were counted by umi_tools with an editing distance threshold of 1.

### scRNA-seq cell analyses

Single-cell analyses were mainly performed with Seurat 3.0^78^, which included cell/gene selection, variance regression, data normalization, multiple sample integration, cell clustering, cluster-level marker gene finding, and data visualization. Three levels of analyses were carried out sequentially: the first was on the whole cells extracted by umi_tools, the second was on the cells excluding red blood cells, and the third was on the subpopulations based on the second-level analyses. In the first-level analyses, only the genes with UMIs larger than 0 in at least three cells were retained, and the remaining cells contained between 300 and 4000 detected genes. The normalization of the expression data for cell clustering was performed by sctransform (SCT) algorithms (https://github.com/ChristophH/sctransform), and 3000 genes were selected for multiple sample integrations based on the ranking scores of the SelectIntegrationFeatures function in Seurat. Before the integration of multiple datasets, several variables (percentage of mitochondria, number of detected genes, number of detected UMIs, and cell cycle scores) were selected for the second nonregularized linear regression. Cell cycle scores were calculated by the CellCycleScoring method in Seurat, which relied on the expression of genes associated with the S phase and G2M phase. The uniform manifold approximation and projection (UMAP) technique was used for high-dimensional gene expression data dimensional reduction, and the shared nearest neighbor (SNN) algorithm was applied for cell clustering. Fifty principal components were extracted for dimensional reduction and cell clustering.

In the second-level analyses, the red blood cells based on the annotation from the first-level analyses were removed from further analyses, and five globin genes (Hba-a1, Hba-a2, Hba-ps4, Hbb-bs, and Hbb-bt) were also removed to avoid contamination. Similar procedures to those used in the first-level analyses were applied. To find conserved cluster-level marker genes, for each sample, the target cluster was compared to other clusters by the Wilcoxon rank-sum test. The *P* values were adjusted for multiple comparisons by Bonferroni correction. The maximal adjusted *P* values for each gene in each cluster among all samples were extracted as the combined adjusted *P* value, and the conserved cluster-level marker genes among different samples were defined as the genes with a combined *P* value less than 0.05.

In the third-level analyses, the four major epididymal cell types (principal cells, basal cells, clear/narrow cells, and halo/T cells) found in the second-level analyses were further independently analyzed. Similar to the first two levels, the same four variables (percentage of mitochondria, number of detected genes, number of detected UMIs, and cell cycle scores) were regressed based on the values calculated for each subpopulation. A higher clustering resolution (0.1 compared to 0.05 for the first two levels of analyses) was chosen for SNN cell clustering.

For visualization, the raw UMI was normalized to reads per million (RPM) and then scaled by the natural logarithm by adding 1 to each to avoid inputs with a value of 0. This normalized and scaled value was named log1pRPM for brevity.

### Cell type annotation

Epididymal cell-type annotation was based on known epididymal cell marker genes that we collected from previously published studies. The average expression level of each cell cluster was calculated based on log1pRPM. Twenty-six known epididymal cell marker genes were collected based on previous studies (Supplementary Table S1), and the average log1pRPM was examined for each cell cluster of those marker genes.

### Epididymis segmentally DEGs

First, segmental-differential (difference among caput, corpus, and cauda segments) analysis (segmental DEGs) was carried out on each cell cluster in each sample. In detail, the nonparametric Kruskal-Wallis test was used to calculate the *P* values of the segmental difference. The *P* values were further adjusted by Bonferroni correction for multiple comparisons. For each sample, the proportion of cells expressed in each cluster of each segment was calculated, and then the sample-level minimal and segmental-highest proportion was required to be larger than 25% for significant segmental DEGs. The fold change was calculated as the log1pRPM difference between the segment with the highest average expression level and the segment with the lowest average expression level (foldchange = log1pRPM_high-log1pRPM_low = log((1 + RPM_high)/(1 + RPM_low)), and the sample-level minimal fold change was required to be larger than 2 for significant segmental DEGs. Then, the sample-level maximal Bonferroni-adjusted *P* value was set to be less than 0.05 for significant segmental DEGs. Finally, only segmental DEGs that were specifically differentially expressed in one population were retained as final epididymis segmental DEGs.

### Epididymis age-related differentially expressed genes

Similar to segmental DEG analysis, in age-related DEG analysis, a nonparametric Kruskal-Wallis test was applied to test the differential expression between the cells from 56-day-old mice and 42-day-old mice for each cell cluster. For significant age-related DEG selection, the maximal percentage of cells detected in either age group should be larger than 50%, the log1pRPM difference between the two groups should be larger than 1, and the adjusted *P* value should be less than 0.01.

### Gene enrichment analyses

For each subpopulation of epididymis or epididymal principal cells, the subpopulation (or cell cluster)-specific marker genes were selected for gene enrichment analyses, and the analyses were performed by clusterProfiler^79^. Benjamini-Hochberg *P* value adjustment was applied to multiple testing, and GO terms with enrichment adjusted *P* values less than 0.05 were selected. To study the pathways of corpus cauda-enriched mitochondrial genes, the gene names were first converted to NCBI Entrez ID and then submitted to the DAVID online tool (https://david.ncifcrf.gov)^80^.

### Ligand-receptor-based cell-cell communication study

Mouse ligand-receptor interaction information was based on the annotation from CellTalkDB^81^, and the SingleCellSignalR^82^ packages were used for paracrine intercellular network analysis. The default database in SingleCellSignalR was replaced by CellTalkDB using the R package provided by CellTalkDB (scsrctdb-1.0.tar.gz, https://github.com/ZJUFanLab/CellTalkDB).

### Mitochondrion staining

After dissection, the epididymis was fixed in 4% paraformaldehyde (Sangon, Shanghai, China) for 1 h at room temperature. The PFA was removed, and the fixed epididymis was washed in PBS for 30 min at room temperature. The tissues were gradient dehydrated in 5%, 10%, and 30% sucrose solutions (Sangon, Shanghai, China) overnight at 4 °C. The epididymis was embedded in optimum cutting temperature compound (O.C.T., Tissue-Tek® oct compound, Sakura, USA) and frozen in liquid nitrogen. Sections were microtomed on a Leica CM1950 clinical cryostat (Leica Biosystems, Australia) at 8 μm thickness and collected onto adhesion microscope slides (Citotest, Nantong, China). Tissue sections were heat-fixed to slides at 60 °C for at least 1 h, and slides were stored at −20 °C. For mitochondrial staining, the staining solution of MitoTracker® Red CMXRos (Thermo Fisher Scientific Inc., M7512) was prepared from a 1 mM stock (dissolved in DMSO; Sigma-Aldrich, USA), which was diluted in sterile PBS to a final concentration of 70 nM. The frozen sections were thawed at room temperature for 30 min and permeabilized with 0.1% Triton X-100 (Sangon, Shanghai, China) in PBS for 30 min at room temperature. The slides were fully submerged in the staining solution and incubated at room temperature with gentle agitation provided by a shaker for 30 min. The staining solution was aspirated, and the slides were then washed for 5 min in PBS. DAPI staining (2 mg/mL in PBS; Thermo Fisher Scientific Inc., 62247) was performed for 5 min, followed by a 5-min wash in PBST. Finally, the sections were mounted in ProLong™ Gold Antifade Mounting medium (Molecular Probes) and then stitched by an imaging reader (Cytation 1, BioTek). High-magnification images were acquired with a confocal microscope (Leica TCSSP8, Leica). The corresponding statistical data were analyzed with Fiji developed from ImageJ^83^ with gating for cell size and epididymal tube shape to exclude the signals from spermatozoa and nonspecific staining.

### Immunofluorescence

The epididymides were dissected and fixed for at least 24 h by immersion in 4% paraformaldehyde (Sangon, Shanghai, China). The fixed tissues were then processed for paraffin embedding after tissue processing. Then, 5 μm-thick sections were cut from each epididymis, and immunofluorescence analysis was performed. Sections were blocked with blocking solution (5% donkey serum and 0.1% Triton X-100 in 2% BSA) for 60 min at room temperature after antigen retrieval (10 mM sodium citrate buffer, pH 6.0 for 15 min). The specimen was covered with diluted rabbit anti-cytochrome c antibody (1:100, Cell Signaling Technology, 11940S) overnight at 4 °C. Normal rabbit IgG was used as a negative control. After removing the primary antibody and washing three times (5 min each) with PBST, the secondary antibody (Alexa Fluor 568-conjugated donkey anti-rabbit IgG (H + L) highly cross-adsorbed secondary antibody, Invitrogen, 1:500) was added for 1 h at room temperature. The nuclei were stained with DAPI solution (2 mg/mL in PBS; Thermo Fisher Scientific Inc., 62247) for 5 min. All slides were mounted with ProLong™ Gold Antifade Mountant (Molecular Probes) and then analyzed by an imaging reader (Cytation 1, BioTek) and confocal microscopy (Leica TCSSP8, Leica). The corresponding statistical data were analyzed with Fiji with gating for cell size and epididymal tube shape to exclude the signals from spermatozoa and nonspecific staining.

### RNA preparation and real-time PCR of the epididymal segments

To validate the expression of mitochondrial genes in epididymal cells, sperm-depleted dissection of the epididymis was performed according to the previous description^37^. Of note, the epididymis segment (caput, corpus, and cauda) separation followed the conventional illustration^7,84^. The isolated sperm-depleted epididymal segments were stored at −80 °C for subsequent RNA extraction.

The total RNA was extracted by TRIzol (Sigma, USA), and 2 µg of RNA was reverse-transcribed by the PrimeScript^TM^ RT reagent Kit with gDNA Eraser (TaKaRa, Japan) according to the manufacturer’s instructions. Real-time PCR with a standard program was performed on a Roche LightCycler 96 real-time fluorescence quantitative PCR instrument (Roche) using ChamQTMSYBR® qPCR Master Mix (Vazyme, Nanjing, China). All genes in each sample were investigated three times, and data processing was performed based on the ΔΔCt method^85^. The mitochondrial genes and sequences of the primers are provided in Supplementary Table S2. Statistical significance for comparison was determined using one-way ANOVA with post hoc analysis. Values of *P* < 0.05 were considered significant.

## Supplementary information

Supplementary Information

Supplementary Table S3

Supplementary Table S4

Supplementary Table S5

Supplementary Table S6

Supplementary Table S7

Supplementary Table S8

Supplementary Table S9

Supplementary Table S10

## Data Availability

The raw data files of scRNA-seq were deposited in the Gene Expression Omnibus under accession number GSE159713. R code files for the main steps of the analysis are available upon reasonable request. The custom R code used is available at https://github.com/gangcai/mouseEpididymis
