# Supplementary material for: Spatio-temporal landscape of mouse epididymal cells and specific mitochondria-rich segments defined by large-scale single-cell RNA-seq
Source: Cell Discov. 2021 May 18;7:34. doi: 10.1038/s41421-021-00260-7 (PMC8129088; doi:10.1038/s41421-021-00260-7)
Supplement: Supplementary file 1 — Supplementary Information [file 41421_2021_260_MOESM1_ESM.pdf]

Supplementary information

Supplementary Figures S1-S12

Supplementary Tables S1-S10

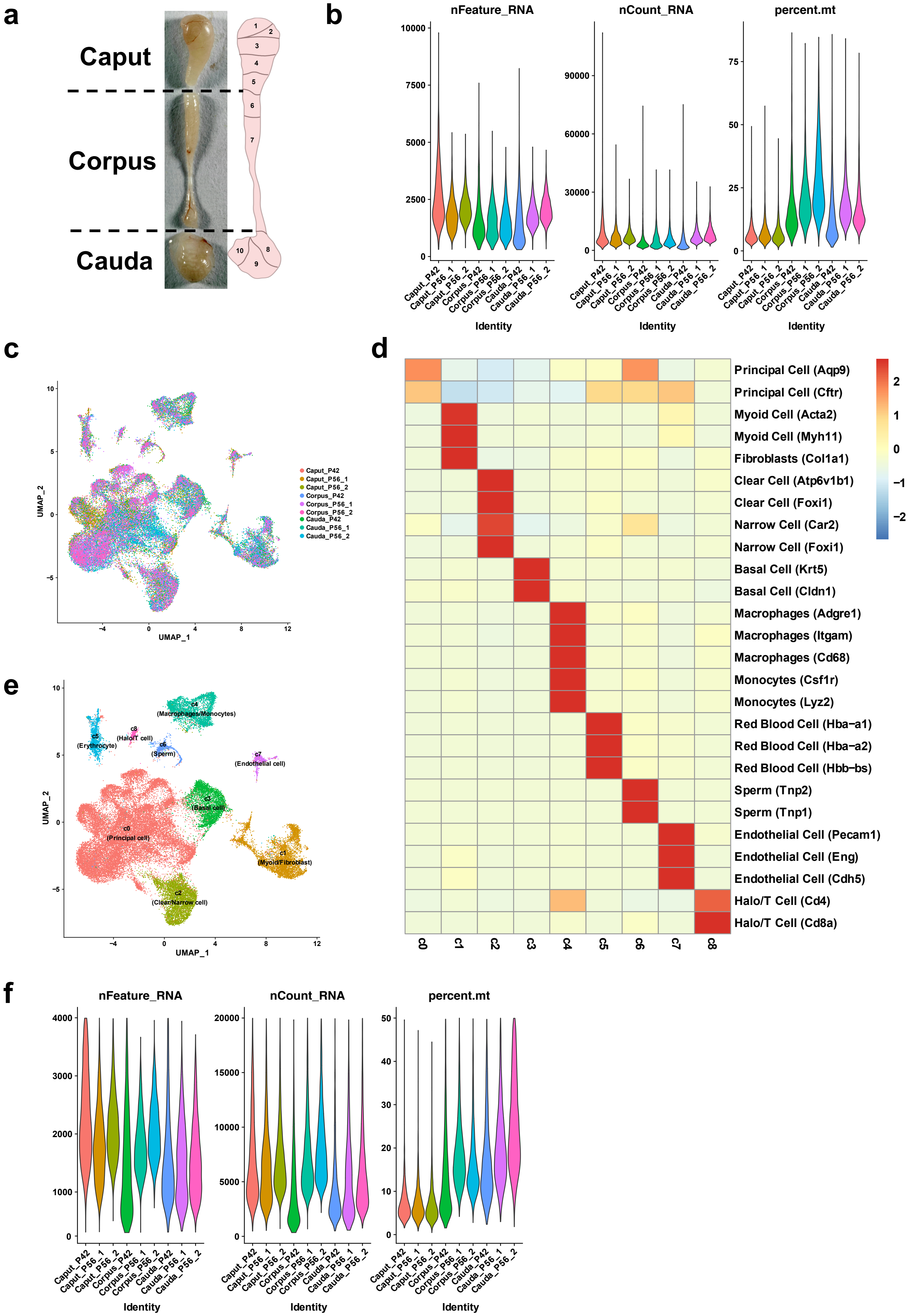
a
Caput
Corpus
Cauda
1
2
3
4
5
6
7
8
9
10
b
nFeature_RNA
nCount_RNA
percent.mt
10000
7500
5000
2500
0
90000
60000
30000
75
50
25
Caput_P42
Caput_P56_1
Caput_P56_2
Corpus_P42
Corpus_P56_1
Corpus_P56_2
Cauda_P42
Cauda_P56_1
Cauda_P56_2
Identity
c
UMAP_1
UMAP_2
d
Principal Cell (Aqp9)
Principal Cell (Cftr)
Myoid Cell (Acta2)
Myoid Cell (Myh11)
Fibroblasts (Col1a1)
Clear Cell (Atp6v1b1)
Clear Cell (Foxi1)
Narrow Cell (Car2)
Narrow Cell (Foxi1)
Basal Cell (Krt5)
Basal Cell (Cldn1)
Macrophages (Adgre1)
Macrophages (Itgam)
Macrophages (Cd68)
Monocytes (Csf1r)
Monocytes (Lyz2)
Red Blood Cell (Hba−a1)
Red Blood Cell (Hba−a2)
Red Blood Cell (Hbb−bs)
Sperm (Tnp2)
Sperm (Tnp1)
Endothelial Cell (Pecam1)
Endothelial Cell (Eng)
Endothelial Cell (Cdh5)
Halo/T Cell (Cd4)
Halo/T Cell (Cd8a)
c0
c1
c2
c3
c4
c5
c6
c7
c8
2
1
0
−1
−2
e
c4 (Macrophages/Monocytes)
c5 (Erythrocyte)
c8 (Halo/T cell)
c6 (Sperm)
c7 (Endothelial cell)
c3 (Basal cell)
c0 (Principal cell)
c1 (Myoid/Fibroblast)
c2 (Clear/Narrow cell)
f
nFeature_RNA
nCount_RNA
percent.mt
4000
3000
2000
1000
20000
15000
10000
5000
50
40
30
20
10
Identity

Supplementary Fig. S1 Sample collection and quality control (QC) for the scRNA-seq datasets. **a** Representative image of dissected mouse epididymis with the caput, corpus and cauda regions. Left: dissected epididymis tissue from a mouse. Right: Conventional segment definition of the mouse epididymis according to the literature. **b** Distribution of the number of genes detected, number of UMIs and percentage of mitochondria (before cell filtering). **c** UMAP visualization of aligned samples. **d** Cell annotation of each epididymal cell population based on cell type-specific marker genes from the literature (log1pRPM values are centred and scaled by row). **e** UMAP visualization of the nine cell clusters identified in the epididymis, including erythrocytes. **f** Distribution of the number of genes detected, number of UMIs and percentage of mitochondria (after cell filtering).

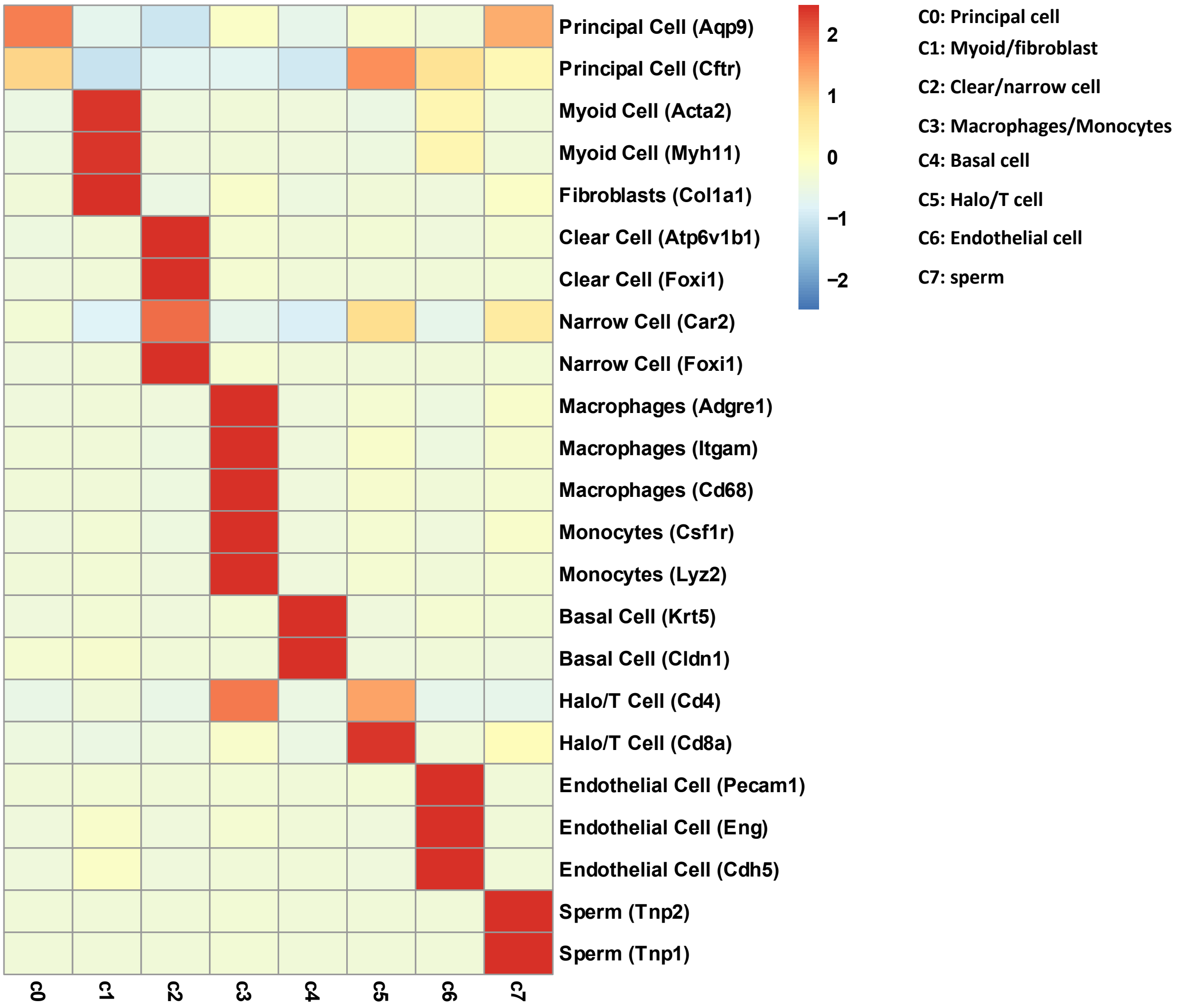

Supplementary Fig. S2 Cell annotation of each epididymal cell population based on cell type-specific marker genes from the literature (log1pRPM are centred and scaled by row).

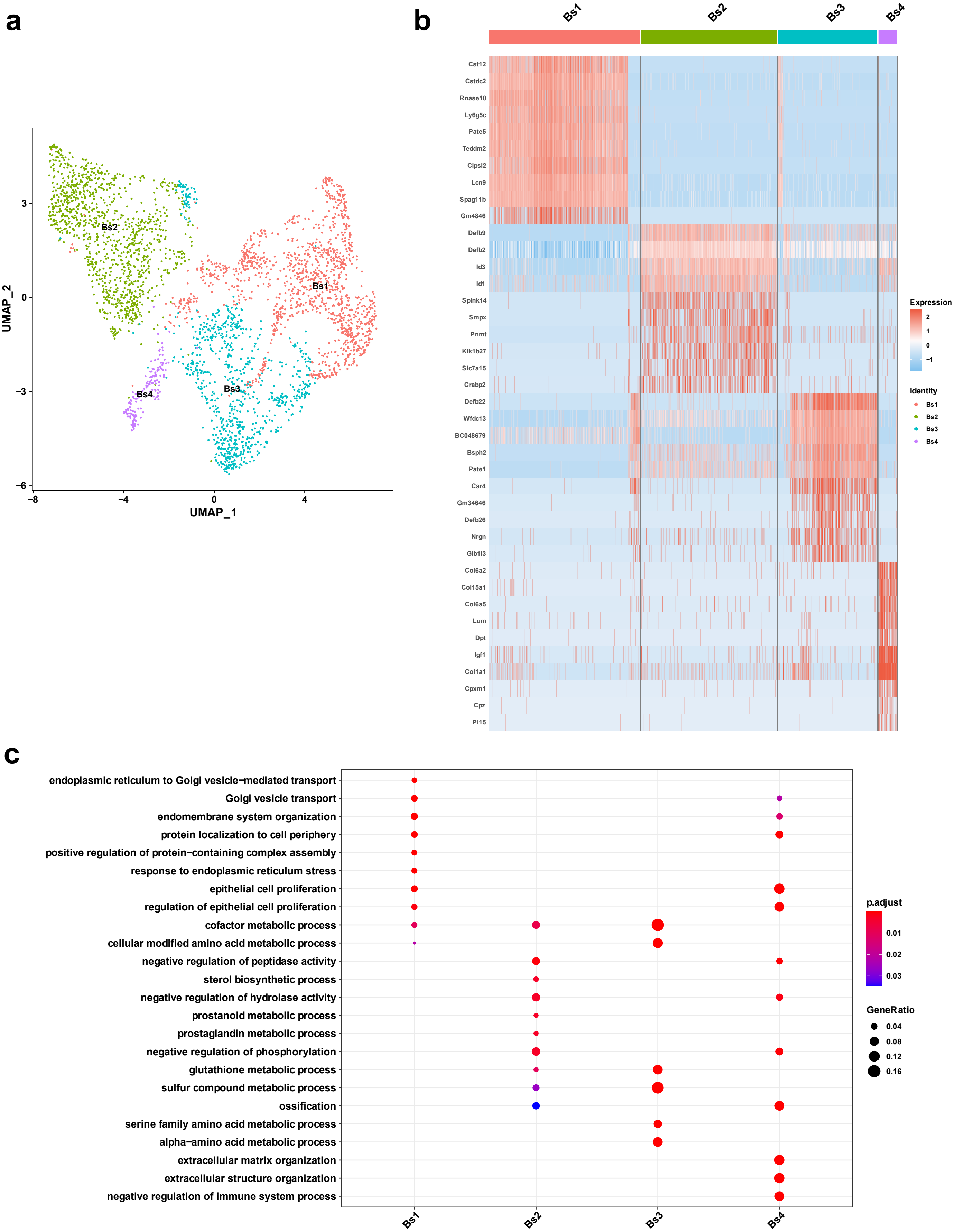

Supplementary Fig. S3 Sub-populations of basal cells. a UMAP visualization of subclusting of basal cells. **b** The top 10 DEGs in each subcluster are displayed by heatmaps. **c** GO enrichment analysis of DEGs for each basal cell subpopulation.

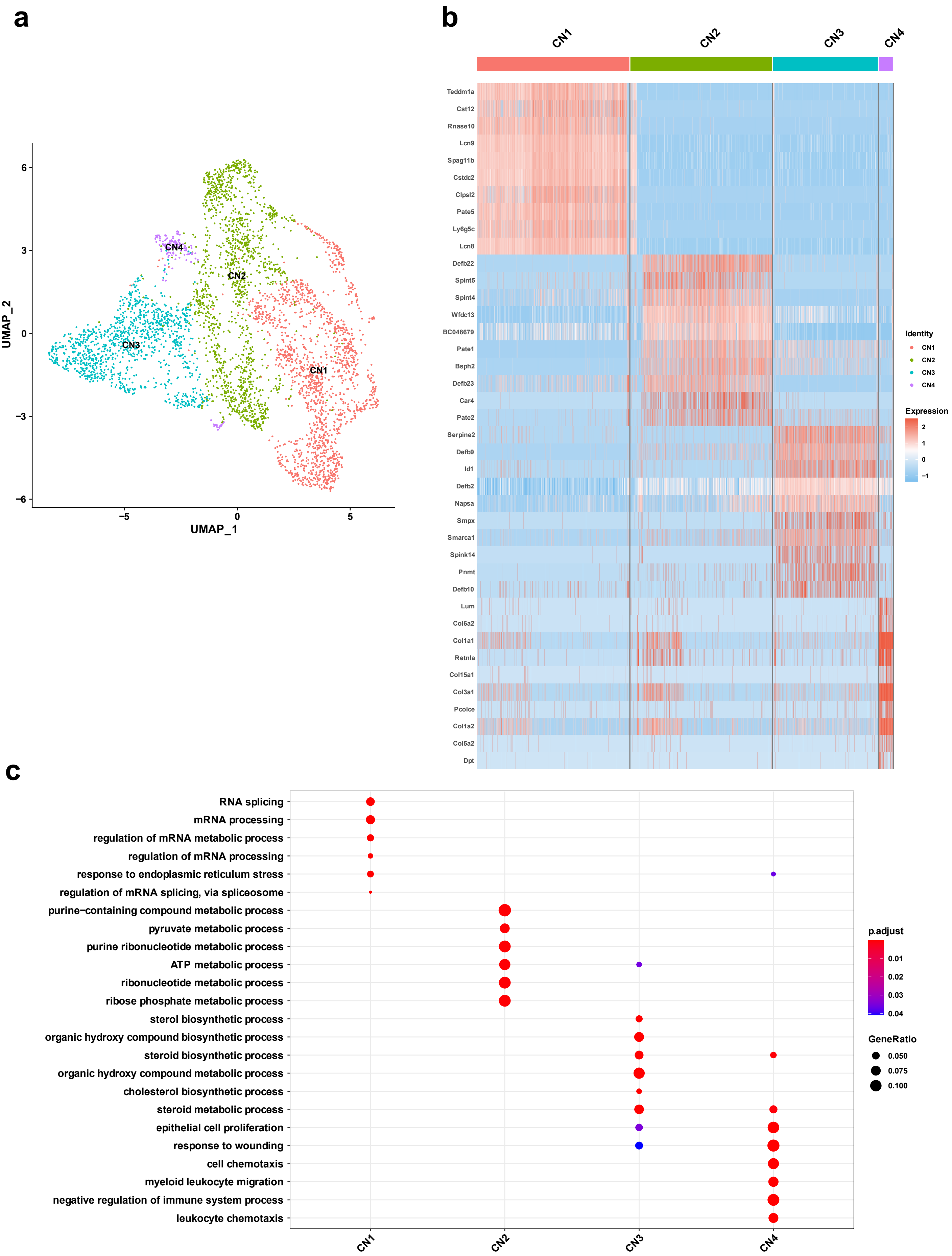

Supplementary Fig. S4 Sub-populations of clear/narrow cells. a UMAP visualization of subclusting of clear/narrow cells. **b** The top 10 DEGs in each subcluster are displayed by heatmaps. **c** GO enrichment analysis of DEGs for each clear/narrow cell subpopulation.

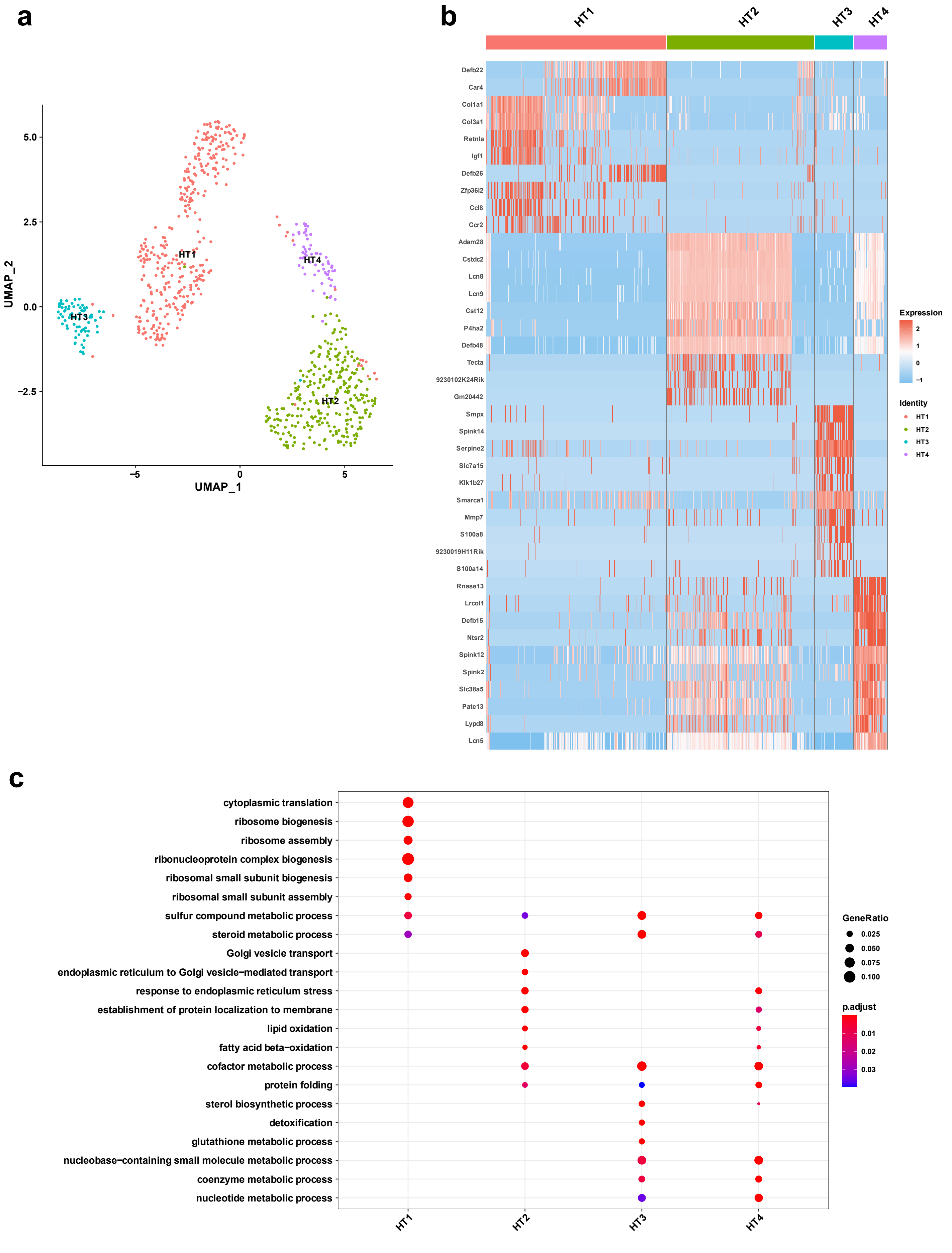

Supplementary Fig. S5 Sub-populations of Halo/T cells. a UMAP visualization of subclusting of Halo/T cells. **b** The top 10 DEGs of each subcluster are displayed by heatmaps. **c** GO enrichment analysis of DEGs for each halo/T cell subpopulation.

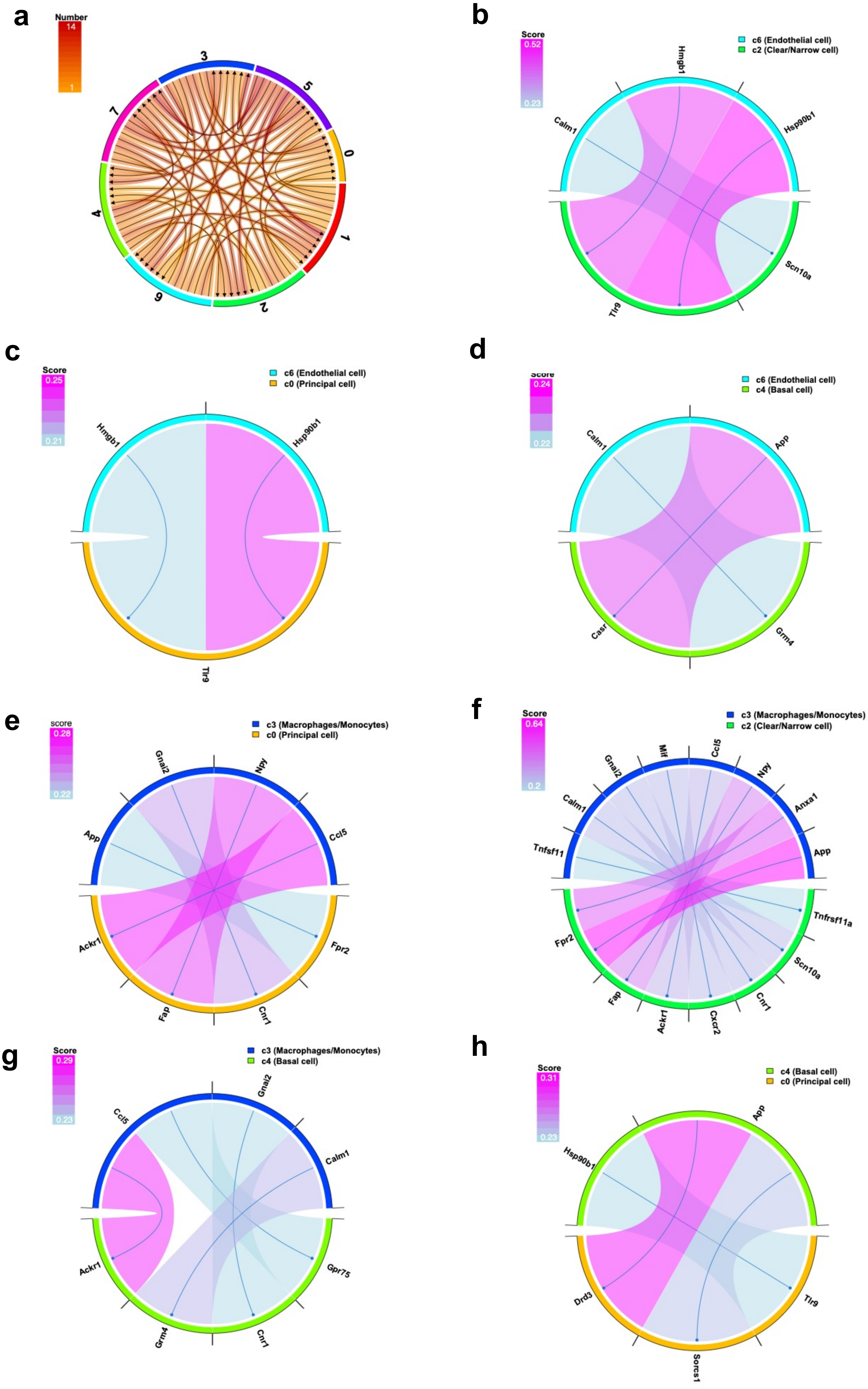
a
Number
14
1
3
5
0
1
2
6
4
7
b
Score
0.52
0.23
c6 (Endothelial cell)
c2 (Clear/Narrow cell)
Hmgb1
Calm1
Hsp90b1
Scn10a
Tlr9
c
Score
0.25
0.21
c6 (Endothelial cell)
c0 (Principal cell)
Hmgb1
Hsp90b1
Tlr9
d
Score
0.24
0.22
c6 (Endothelial cell)
c4 (Basal cell)
Calm1
App
Casr
Grm4
e
score
0.28
0.22
c3 (Macrophages/Monocytes)
c0 (Principal cell)
Gnai2
Npy
App
Ccl5
Ackr1
Fpr2
Fap
Cnr1
f
Score
0.64
0.2
c3 (Macrophages/Monocytes)
c2 (Clear/Narrow cell)
Gnai2
Mif
Ccl5
Npy
Calm1
Anxa1
Tnfsf11
App
Tnfrsf11a
Fpr2
Scn10a
Fap
Ackr1
Cxcr2
Cnr1
g
Score
0.29
0.23
c3 (Macrophages/Monocytes)
c4 (Basal cell)
Ccl5
Gnai2
Calm1
Ackr1
Gpr75
Grm4
Cnr1
h
Score
0.31
0.23
c4 (Basal cell)
c0 (Principal cell)
App
Hsp90b1
Drd3
Tlr9
Sorcs1

Supplementary Fig. S6 Cell-cell communication and potential signalling interactions between epididymal cell types. a Cell-cell communication networks between epididymal cell types. The surrounding numbers are the labels of cell clusters. 0: Principal cell, 1: myoid/fibroblast, 2: clear/narrow cell, 3: macrophage/monocytes, 4: basal cell, 5: Halo/T cell, 6: endothelial cell, 7: sperm. (b-d) Ligand-receptor analysis between endothelial cells (c6) and epididymal epithelial cell types. (e-g) Ligand-receptor analysis between macrophage/monocyte cells (c3) and epididymal epithelial cell types. (h) Ligand-receptor analysis between basal cells and principal cells.

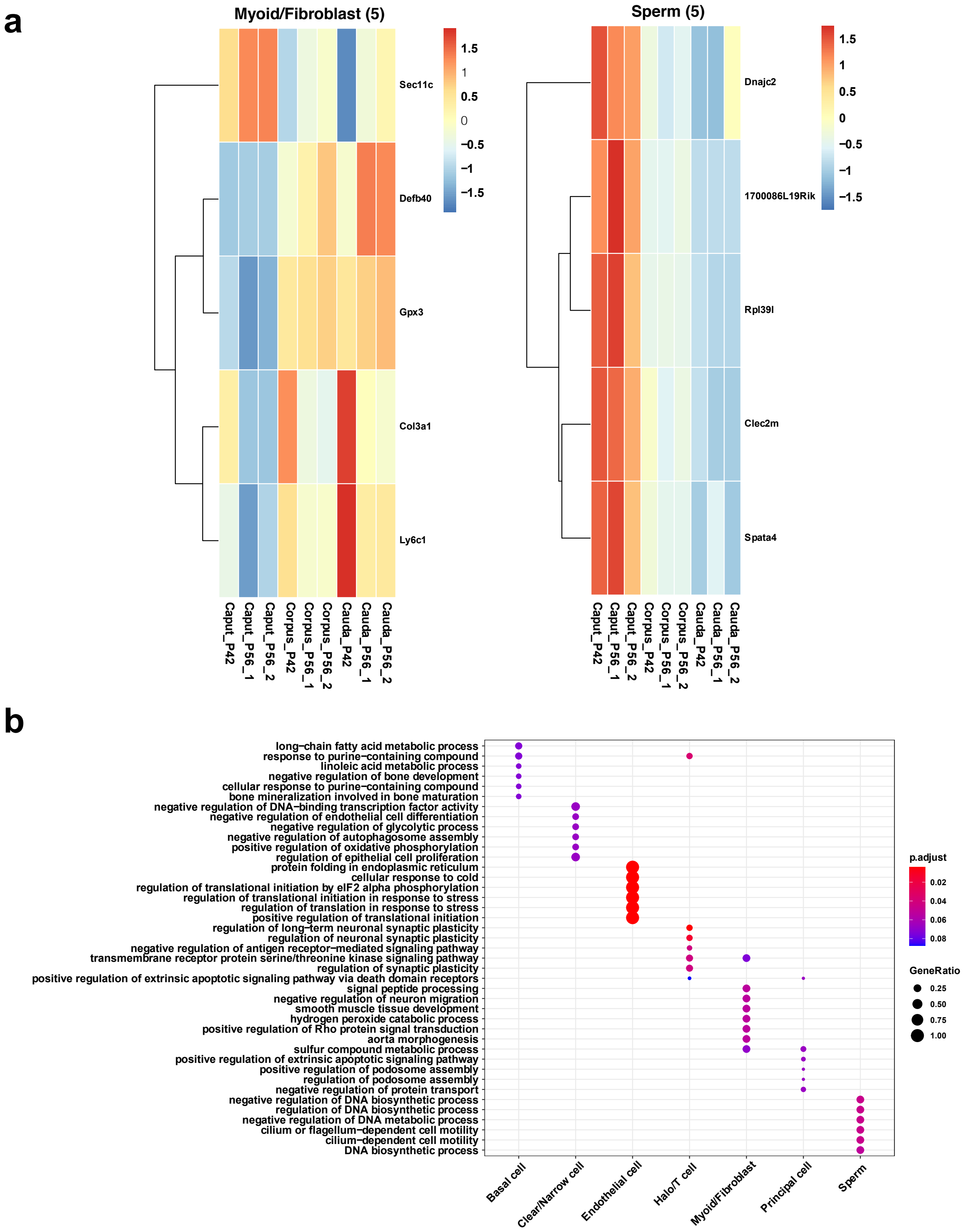

Supplementary Fig. S7 Segmental DEGs of the cells in the mouse epididymis. a Heatmaps showing the segmental DEG expression pattern of each nonepithelial cell in three epididymal regions. **b** GO term analysis.

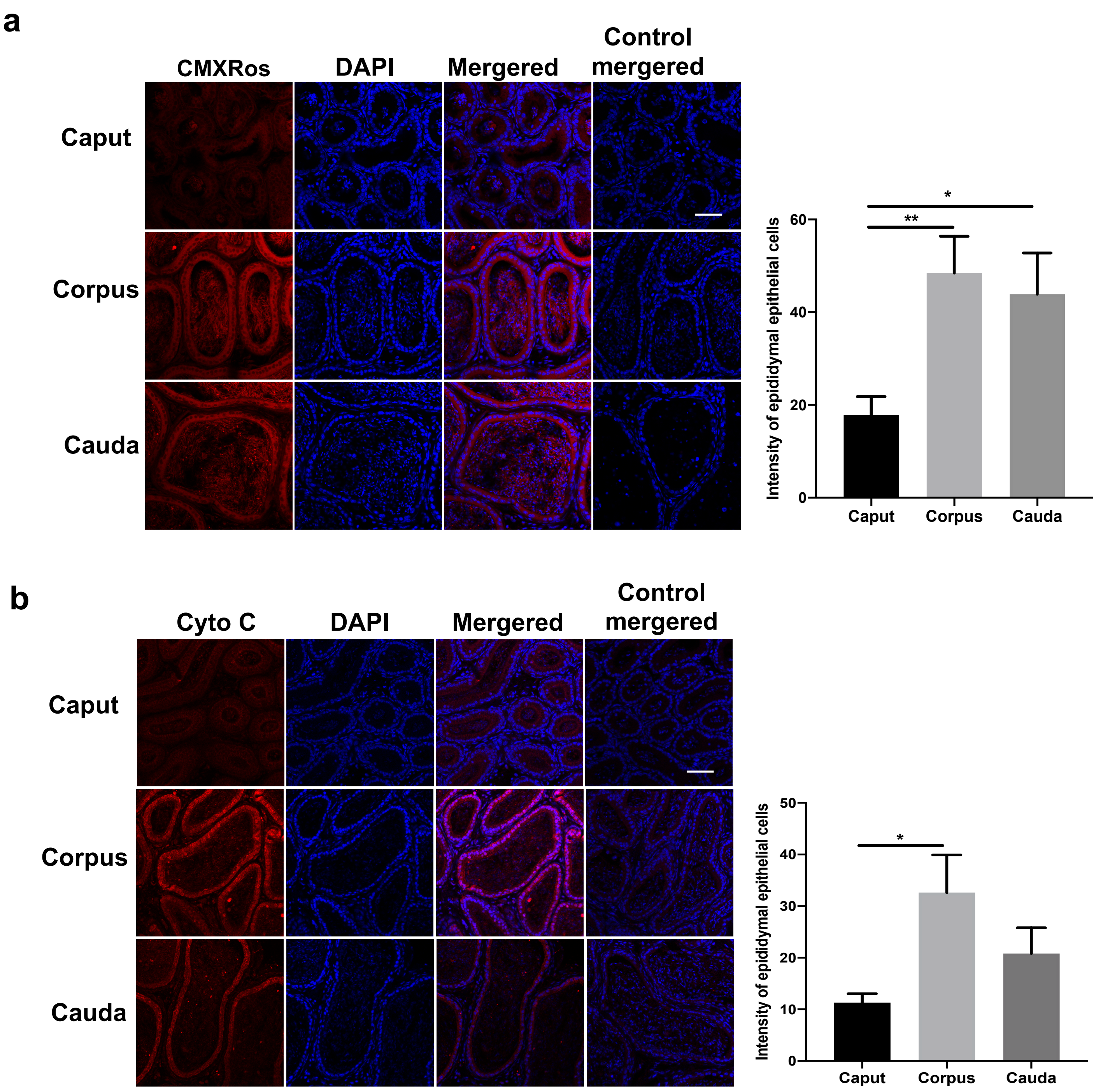

Supplementary Fig. S8 Characterization of the mitochondrial distribution in different epididymal regions in P42 mice. a Representative images of CMXRos staining in the caput, corpus and cauda epididymis of 42-day-old mice. Scale bar=50 μm. Red: CMXRos, Blue: DAPI for nuclear count staining. Right: Corresponding statistics of CMXRos staining in the segmental epididymis. *, P<0.05; **, P<0.01. **b** Representative images of cytochrome c staining in the caput, corpus and cauda epididymis of 42-day-old mice. Scale bar=50 μm. Red: cytochrome c (Cyto C), Blue: DAPI for nuclear count staining. Right: Corresponding statistics of cytochrome c staining in the segmental epididymis. *, P<0.05.

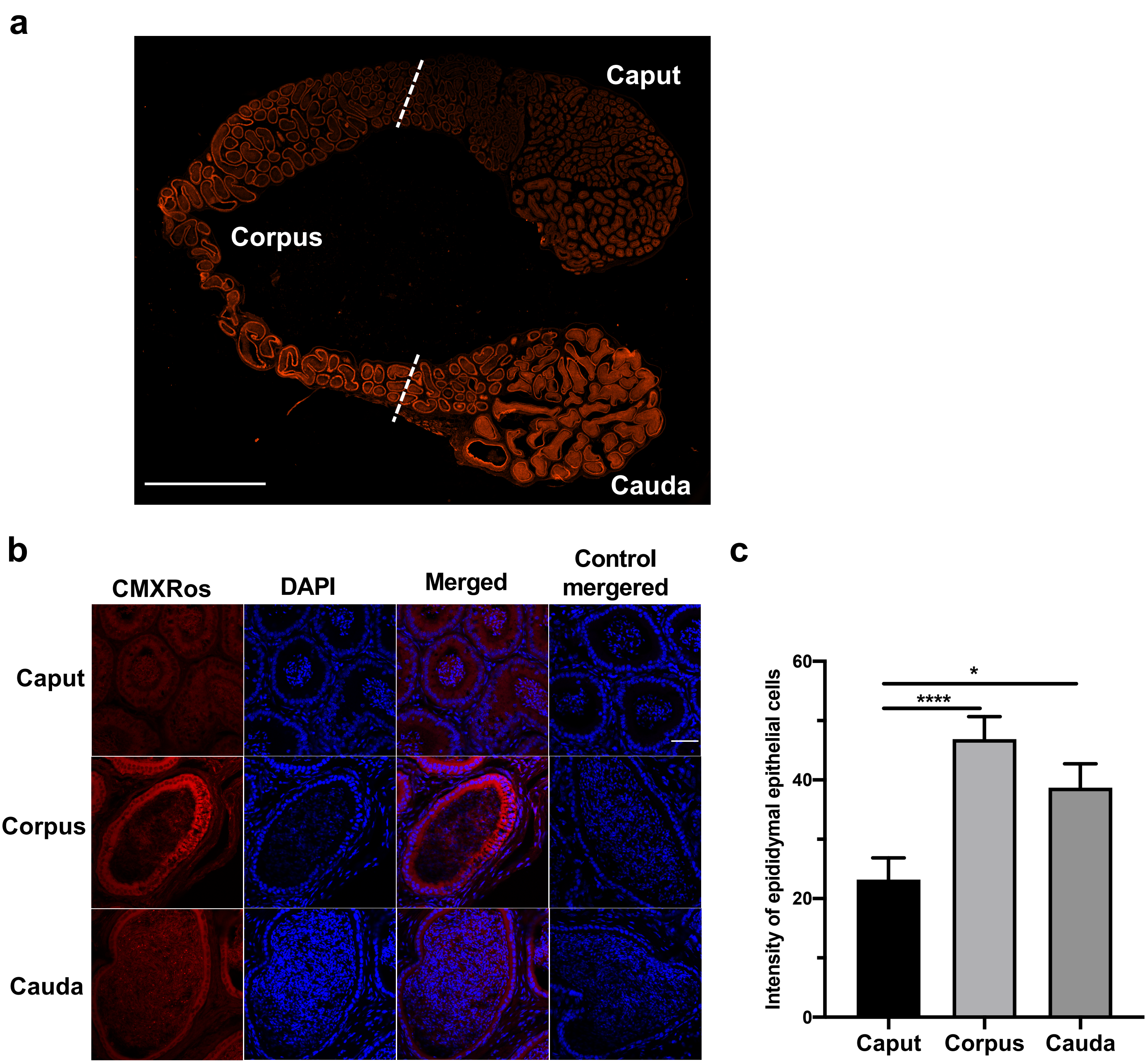

Supplementary Fig. S9 Characterization of the mitochondrial distribution in different epididymal regions in P56 mice. a Stitched images of MitoTracker staining of the P56 epididymis. Scale bar=1 mm. Red: CMXRos for mitochondria staining. **b** Representative images of CMXRos staining in the caput, corpus and cauda epididymis. Scale bar=50 μm. Red: CMXRos, Blue: DAPI for nuclear count staining. **c** Corresponding statistic of CMXRos staining in the segmental epididymis. *, P<0.05; ****, P<0.0001.

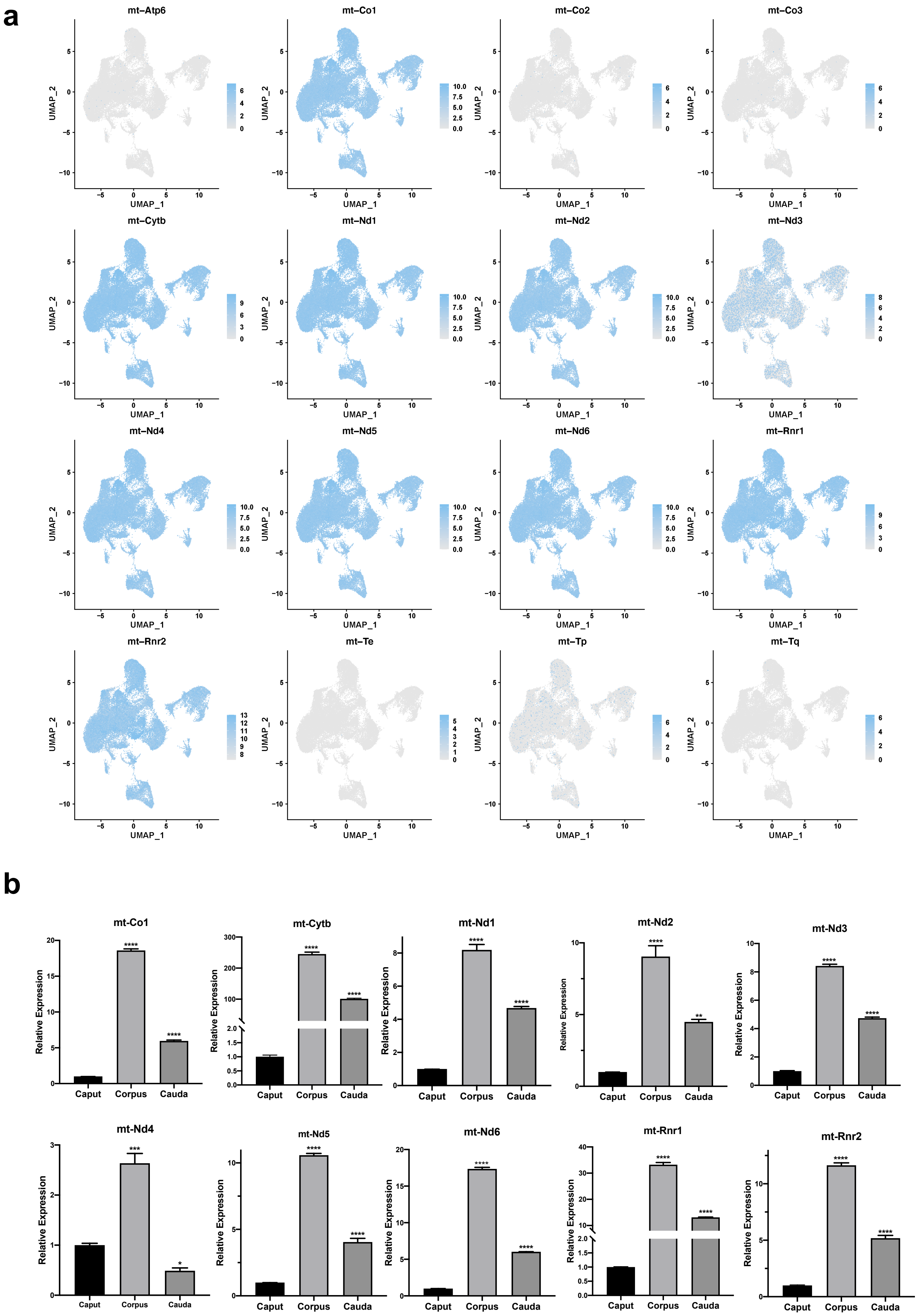
a
mt–Atp6
mt–Co1
mt–Co2
mt–Co3
mt–Cytb
mt–Nd1
mt–Nd2
mt–Nd3
mt–Nd4
mt–Nd5
mt–Nd6
mt–Rnr1
mt–Rnr2
mt–Te
mt–Tp
mt–Tq
UMAP_1
UMAP_2
b
mt-Co1
mt-Cytb
mt-Nd1
mt-Nd2
mt-Nd3
mt-Nd4
mt-Nd5
mt-Nd6
mt-Rnr1
mt-Rnr2
Relative Expression
Caput
Corpus
Cauda

Supplementary Fig. S10 Characterization of mitochondrial transcript genes in epididymal regions. a UMAP plots of mitochondrial transcript genes in the indicated cell clusters (colour values are log1pRPM). **b** Validations of the selected mitochondrial transcript gene in the sperm-depleted epididymal segments by qPCR analysis. The statistical significance was corpus or cauda vs. caput. *, $P<0.05$; ***, $P<0.001$; ****, $P<0.00001$.

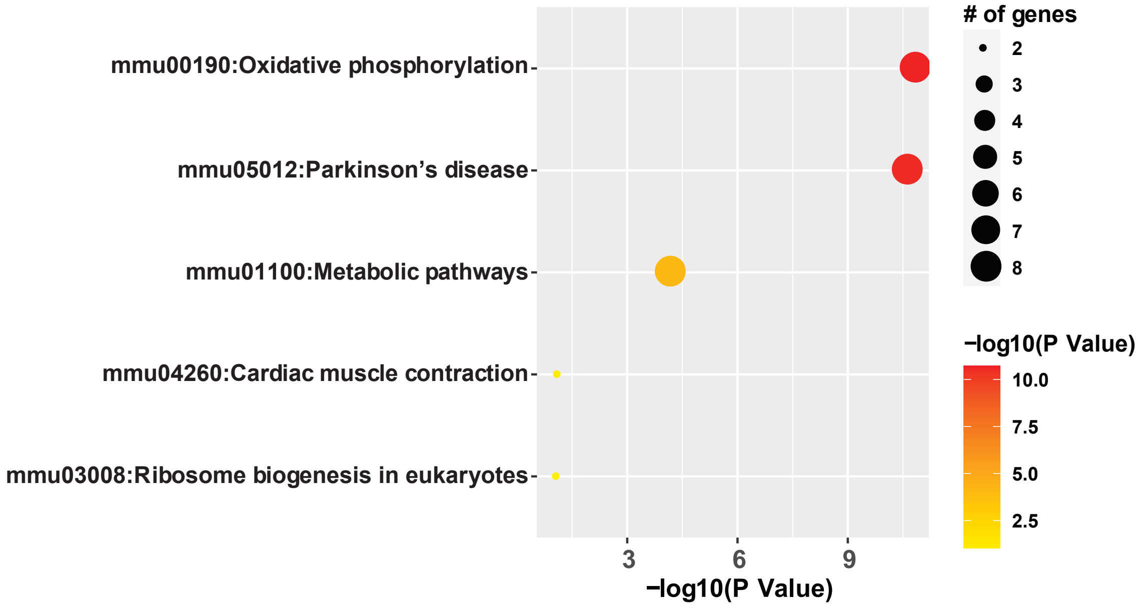

Supplementary Fig. S11 KEGG analysis for the mt-genes.

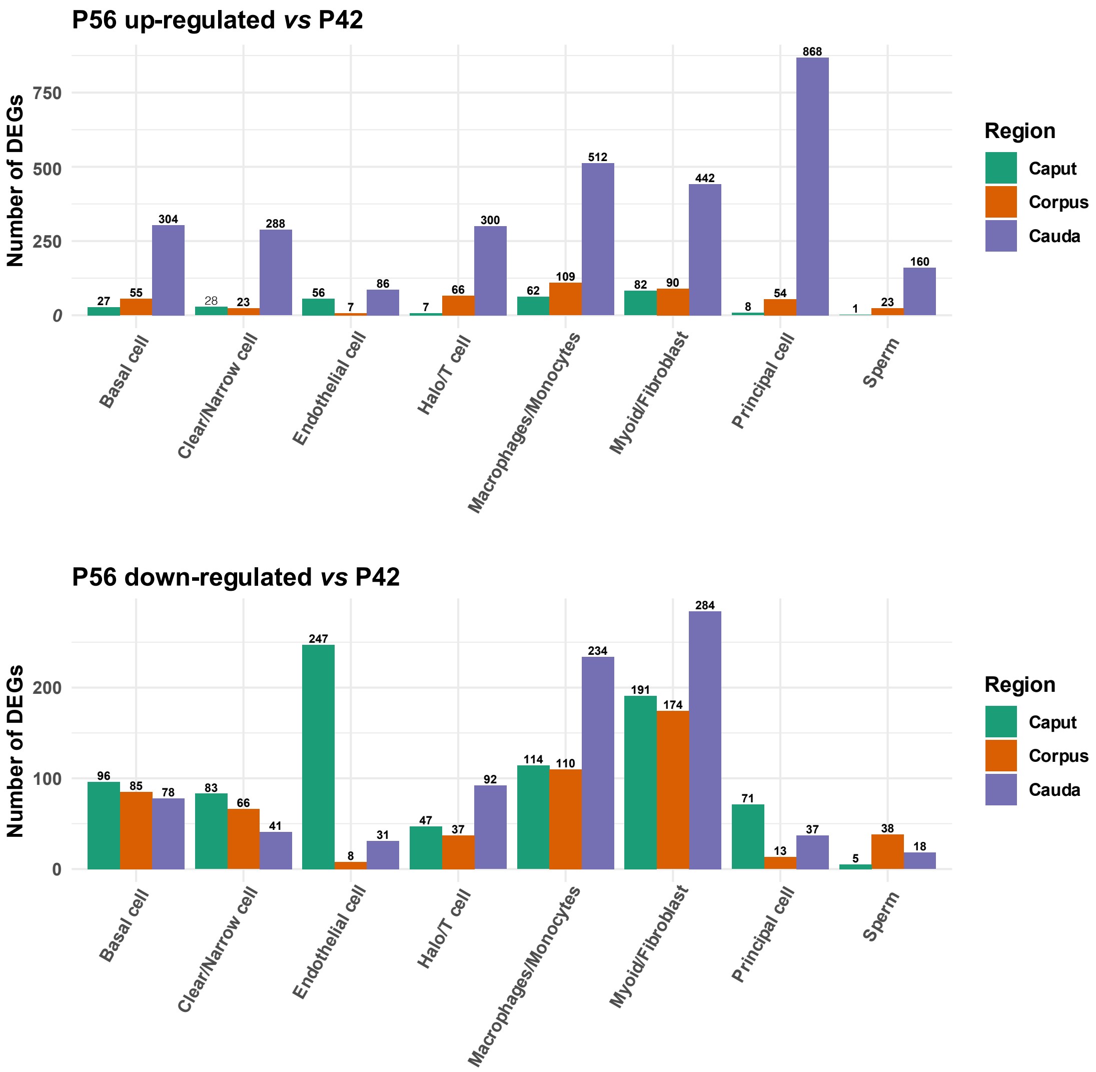

Supplementary Fig. S12 The bar plot shows the DEGs (P56 *vs* P42) of each cell cluster expressed in the different epididymal regions.

Supplementary Table S1. Marker gene of epididymal cells in the literature.

	Cell Type	Gene Full Name	Gene Symbol	Reference
Epididymal Epithelial cells	Principal cell	Aquaporin 9	AQP9	[1-3]
		CF Transmembrane Conductance Regulator	CFTR	[4, 5]
	Basal cell	Keratin 5	Krt5	[1, 2, 6, 7]
		Claudin-1	Cldn1	[7, 8]
	Clear cell	V-ATPase B1 subunit	Atp6v1b1	[2-4, 7]
		Forkhead Box I1	Foxi1	[9]
	Narrow cell	Carbonic anhydrase II	Car2	[9]
		Forkhead Box I1	Foxi1	[9]
		CD4 antigen	Cd4	[10]
		CD8 antigen, alpha chain	Cd8a	[10]
Non-epithelial cells	Spermatozoa	Transition Protein 2	Tnp2	[11]
		Transition Protein 1	Tnp1	[11, 12]
	Myoid cell	Actin Alpha 2, Smooth Muscle	Acta2	[13-15]
		Myosin Heavy Chain 11	Myh11	[16]
	Fibroblasts	Collagen Type I Alpha 1 Chain	Col1a1	[13]
	Macrophages	Adhesion G Protein-Coupled Receptor E1	F4/80 (Adgre1)	[1, 6, 17-19]
		Integrin subunit alpha M	Itgam	[18-20]
		CD68 Molecule	CD68	[17, 19, 20]
	Monocytes	Colony stimulating factor 1 receptor	CD115, Csf1r	[13]
		Lysozyme 2	Lyz2	[13]
	Endothelial cells	Platelet And Endothelial Cell Adhesion Molecule 1	Pecam1	[21, 22]
		Endoglin	Eng	[21, 22]
		Cadherin 5	Cdh5	[13, 21, 22]
	Erythrocyte (Red blood cells)	Hemoglobin Subunit Alpha 1	Hba-a1	[21]
		Hemoglobin Subunit Alpha 2	Hba-a2	[21]
		hemoglobin, beta adult s chain	Hbb-bs	[21]

Reference

1. Zhu, W., et al., *Pattern recognition receptor-initiated innate antiviral responses in mouse epididymal epithelial cells.* J Immunol, 2015. **194**(10): p. 4825-35.
2. Carvajal, G., et al., *Impaired male fertility and abnormal epididymal epithelium differentiation in mice lacking CRISP1 and CRISP4.* Sci Rep, 2018. **8**(1): p. 17531.
3. Krapf, D., et al., *CSrc is necessary for epididymal development and is incorporated into sperm during epididymal transit.* Developmental Biology, 2012. **369**(1): p. 43-53.
4. Shum, W.W., et al., *Regulation of luminal acidification in the male reproductive tract via cell-cell crosstalk.* J Exp Biol, 2009. **212**(Pt 11): p. 1753-61.
5. Pietrement, C., et al., *Role of NHERF1, Cystic Fibrosis Transmembrane Conductance Regulator,*

and cAMP in the Regulation of Aquaporin 9. Journal of Biological Chemistry, 2008. **283**(5): p. 2986-2996.

6. Shum, W.W., et al., *Epithelial basal cells are distinct from dendritic cells and macrophages in the mouse epididymis.* Biol Reprod, 2014. **90**(5): p. 90.
7. Shum, W.W., et al., *Transepithelial projections from basal cells are luminal sensors in pseudostratified epithelia.* Cell, 2008. **135**(6): p. 1108-17.
8. Shum, W.W.C., et al., *Regulation of luminal acidification in the male reproductive tract via cell-cell crosstalk.* Journal of Experimental Biology. **212**(11): p. 1753-1761.
9. Blomqvist, S.R., et al., *Epididymal expression of the forkhead transcription factor Foxi1 is required for male fertility.* EMBO J, 2006. **25**(17): p. 4131-41.
10. *Distribution of immune cells in the epididymis of the aging Brown Norway rat is segment-specific and related to the luminal content.* 1999. **61**(3): p. 705-14.
11. Hermann, B.P., et al., *The Mammalian Spermatogenesis Single-Cell Transcriptome, from Spermatogonial Stem Cells to Spermatids.* Cell Rep, 2018. **25**(6): p. 1650-1667 e8.
12. Wang, M., et al., *Single-Cell RNA Sequencing Analysis Reveals Sequential Cell Fate Transition during Human Spermatogenesis.* Cell Stem Cell, 2018. **23**(4): p. 599-614 e4.
13. Kalluri, A.S., et al., *Single-Cell Analysis of the Normal Mouse Aorta Reveals Functionally Distinct Endothelial Cell Populations.* Circulation, 2019. **140**(2): p. 147-163.
14. Xie, T., et al., *Single-Cell Deconvolution of Fibroblast Heterogeneity in Mouse Pulmonary Fibrosis.* Cell Rep, 2018. **22**(13): p. 3625-3640.
15. Guo, J., et al., *The adult human testis transcriptional cell atlas.* Cell Res, 2018. **28**(12): p. 1141-1157.
16. Rebourcet, D., et al., *Sertoli cells control peritubular myoid cell fate and support adult Leydig cell development in the prepubertal testis.* Development, 2014. **141**(10): p. 2139-49.
17. Mould, K.J., et al., *Single cell RNA sequencing identifies unique inflammatory airspace macrophage subsets.* JCI Insight, 2019. **4**(5).
18. Zimmerman, K.A., et al., *Single-Cell RNA Sequencing Identifies Candidate Renal Resident Macrophage Gene Expression Signatures across Species.* J Am Soc Nephrol, 2019. **30**(5): p. 767-781.
19. DeFalco, T., et al., *Macrophages Contribute to the Spermatogonial Niche in the Adult Testis.* Cell Rep, 2015. **12**(7): p. 1107-19.
20. Masaki and T., *Heterogeneity of antigen expression explains controversy over glomerular macrophage accumulation in mouse glomerulonephritis.* Nephrology Dialysis Transplantation, 2003. **18**(1): p. 178-181.
21. Kalucka, J., et al., *Single-Cell Transcriptome Atlas of Murine Endothelial Cells.* Cell, 2020. **180**(4): p. 764-779 e20.
22. Lukowski, S.W., et al., *Single-Cell Transcriptional Profiling of Aortic Endothelium Identifies a Hierarchy from Endovascular Progenitors to Differentiated Cells.* Cell Rep, 2019. **27**(9): p. 2748-2758 e3.

Supplementary Table S2. Primers for mitochondrial genes.

Gene	The Sequence of Primers	Gene	The Sequence of Primers
β-actin	F: GGCTGTATTCCCCTCCATCG<br>R: CCAGTTGGTAACAATGCCATGT	mt-Co1	F: GCGGGAATAGTGGGTACTGC<br>R: CGGCTAGAGGTGGGTAGACT
mt-Rnr2	F: CTGCCTGCCCAGTGACTAAA<br>R: GACCCTCGTTTAGCCGTTCA	mt-Rnr1	F: TCTTCGGCGTAAAACGTGTC<br>R: TACCGCCAAGTCCTTTGAGT
mt-Tp	F: GAATACCAGCTTTGGGTGCTG<br>R: AAGGAGCTACTCCCCACCA	mt-Co2	F: TTGGTCTACAAGACGCCACA<br>R: TTGGCAGAACGACTCGGTTA
mt-Cytb	F: AAAGCCACCTTGACCCGATT<br>R: AGGCTTCGTTGCTTTGAGGT	mt-Co3	F: TAACCCTTGGCCTACTCACCA<br>R: TGTGGTGGCCTTGGTAGGTT
mt-Atp6	F: TCCCAATCGTTGTAGCCATCA<br>R: AGACGGTTGTTGATTAGGCGT	mt-Nd1	F: GCATCTTATCCACGCTTCCG<br>R: ATGTATGGTGGTACTCCCGC
mt-Nd2	F: ATCCTCCTGGCCATCGTACT<br>R: ATCAGAAGTGGAATGGGGCG	mt-Nd4	F: ACCCGATGAGGGAACCAAAC<br>R: TCTCGTGTGTGTGAGGGTTG
mt-Nd3	F: GTTGCATTCTGACTCCCCCA<br>R: GGTAGACGTGCAGAGCTTGT	mt-Nd5	TTCCACCCCCTCACGACTAA<br>TGTCGTTTTGGGTGAGAGCA
mt-Nd6	F: GGGGGATGTTGGTTGTGTTTG<br>R: TATTGCCGCTACCCCAATCC		

Supplementary Table S3. Read mapping and single-cell statistics.

Supplementary Table S4. Cluster-level conserved marker genes for the eight epididymal cell populations identified.

Supplementary Table S5. Information about segmental and age-related DEGs.

Supplementary Table S6. Marker genes of subpopulations for principal, basal, clear/narrow, and halo/T cells.

Supplementary Table S7. Details of the GO enrichment analysis.

Supplementary Table S8. Information about the ligand-receptor analysis.

Supplementary Table S9. Information about the average mitochondrial percentage.

Supplementary Table S10. GO analysis of the subpopulations of principal cells.
